# The Influence of Xanthan Gum and Guar Gum Biopolymers on the Geotechnical Properties of Three Different Soils

**DOI:** 10.3390/polym18162006

**Published:** 2026-08-17

**Authors:** Çiğdem Ceylan

**Affiliations:** Faculty of Engineering and Natural Sciences, Civil Engineering, Malatya Turgut Ozal University, 44000 Malatya, Turkey; cigdem.ceylan@ozal.edu.tr; Tel.: +90-(422)-846-12-55

**Keywords:** biopolymer, zeolitic silty soil, xanthan gum, guar gum, shear strength, hydraulic conductivity

## Abstract

This study investigates the macromolecular interaction mechanisms between linear-anionic xanthan gum (XG) and branched-nonionic guar gum (GG) biopolymers in three mineralogically distinct soils: Bentonite Clay (BC), Zeolite Silty Soil (ZS), and Red Clay (RC). Mıxtures were prepared by dry mixing of soil powders with biopolymer powders at designated ratios (0%, 1%, 2%, 3%, and 4% by dry weight). The prepared mixtures were characterized using X-Ray Diffraction (XRD), X-Ray Fluorescence (XRF), Scanning Electron Microscopy (SEM), and standard compaction and shear strength tests. The results show that geotechnical macro-behavior is primarily influenced by polymer chain conformation and mineral interfacial reactions. In ZS-XG mixture, hydraulic conductivity increased approximately 26-fold (from 0.107 × 10^−9^ to 2.83 × 10^−9^ m/s), a phenomenon attributed to the Donnan electrostatic exclusion effect, where linear anionic XG chains repel zeolite surfaces and generate low-friction macro-flow paths. Conversely, the addition of GG to RC formed a strongly interconnected hydrogel network through hydrogen bonding with trivalent iron and magnesium oxides, resulting in a 20.8% increase in cohesion (up to 70.05 kPa). In contrast, GG addition decreased cohesion in BC and ZS. These findings confirm that sustainable biopolymer-based soil remediation depends on customizing the polymer morphology according to the properties of the soil. In engineering applications, ZS-XG mixtures should be evaluated for drainage projects requiring high permeability, whereas the RC-GG4 mixture should be considered a primary option for infiltration barriers (e.g., landfill liners) requiring low permeability and high cohesion.

## 1. Introduction

Accurate characterization of the physical and mechanical properties of soil is essential for effective engineering design and geotechnical applications. The primary objective of soil improvement methods, which employ various physical, chemical, and mechanical techniques, is to address engineering challenges such as enhancing soil bearing capacity, controlling compressibility, reducing swelling potential and permeability, mitigating liquefaction risk, and improving plastic properties. For soils exhibiting insufficient bearing capacity, high settlement potential, or low shear strength, it is necessary to select and implement appropriate soil improvement methods to satisfy engineering requirements [1,2]. The selection and application of these techniques depend on the soil’s inherent properties and specific project needs. Chemical additives, including lime, sodium silicate, epoxy, biopolymers, solid waste, rubber, and polyurethane, are commonly utilized. However, cementitious binders present notable environmental concerns due to their toxicity, high carbon emissions, and disruption of natural ecosystems [3,4,5]. Consequently, there is growing interest in sustainable, environmentally friendly engineering practices, and research into alternative binder systems and chemical improvement approaches with reduced environmental impact has accelerated [6,7,8]. Biopolymers derived from non-food agricultural products are being explored for their sustainability and environmental friendliness, particularly in soil improvement applications, with promising results [9]. Research indicates that biopolymers possess advantageous properties such as high shear resistance, elevated viscosity, pseudoelasticity, and stability across a broad pH and temperature range, which can be effectively utilized in soil improvement [10].

Microbial geotechnology is a developing subfield of geotechnical engineering concerned with the use of microbiological processes in engineering applications [11,12]. The use of biopolymers in problematic soils has increased significantly over the last decade among researchers seeking solutions to minimize environmental concerns [13,14,15]. These materials are considered an alternative to traditional chemical additives due to their environmental friendliness. They can increase overall strength by enhancing cohesion through the gel structure they create between soil grains [9,16,17]. It has been reported that soil mixtures containing small amounts of biopolymer exhibit greater strength than cement [7]. Different effects can occur depending on the type and amount of biopolymer used in soil improvement, as well as its reaction with temperature and water [18].

Biopolymers have been shown to be effective at stabilizing weak soils, especially those such as mining waste. Biopolymers such as Xanthan Gum (XG) and Guar Gum (GG) form a gel-like structure by bringing soil particles together, thus increasing cohesion and stability within the soil [10,19]. In clay-rich soils, they interact with the soil through hydration, swelling, and electrostatic attraction, thereby increasing particle bonding and contributing to increased shear strength [20].

Previous studies have shown that XG and GG biopolymers significantly improve soil engineering properties [18,21,22]. Improvement applications using XG and GG in cohesive and non-cohesive soils have been found suitable by many researchers. However, it is increasingly understood that the performance of biopolymers is critically dependent not only on their type and dosage, but also on factors such as the mineralogical structure, chemical surface properties, and cation exchange capacity of the soil used [10,19]. For example, it is thought that biopolymers may exhibit different interaction mechanisms in montmorillonite-rich bentonitic clays than in kaolinitic or porous zeolitic soils. In this context, comparative investigation of the behavior of XG and GG on different soil types is of great importance for the effective and predictable use of these materials. Zeolite is included in this study not as a clay mineral in the strict sense, but as a representative of highly porous aluminosilicate materials.

XG, one of the most commonly used biopolymers in geotechnical applications such as soil stabilization, mining exploration, erosion control, and permeability control, is produced by the bacterium Xanthomonas Campestris and is also used in food and pharmaceutical applications [1,23,24]. One of its important properties is its solubility in hot and cold water, as well as its ability to increase the viscosity of the environment in which it is located [22]. It has been reported that permeability decreases with XG use [25,26,27]. In addition, it has been reported to exhibit pH sensitivity and high shear strength even at low concentrations [22]. It has been stated that the strength of the soil is significantly increased with the use of XG, the resistance to wetting and drying cycles is increased, and significant improvement is achieved against soil erosion [10,17]. It has been stated that, under suitable conditions, the strength and water resistance of the soil can be increased by creating network structures between soil grains through hydrogen bonding and cation bridging upon the addition of XG [12,21]. It is stated that XG improves the strength and compressibility of clays such as kaolinite and bentonite by forming hydrogels that fill pore spaces [21]. The basic properties of XG and GG used in the studies are given in Table 1.

Most existing studies have concentrated on low-plasticity sands or silts, and the effects of XG on soils with varying mineralogical properties, such as smectite and zeolite, have not been systematically compared. Furthermore, it is not yet established how the widely recognized positive impact of XG on impermeability is influenced by structures with high internal porosity, such as zeolite.

GG is a polysaccharide obtained from the seeds of the guar plant, also known as Cyamopsis tetragonoloba. It provides a colloidal, thixotropic dispersion by forming extremely strong chemical bonds and is highly viscous [10]. Its use as a soil stabilizer is particularly evaluated in the improvement of problematic swelling soils. For example, GG is quite effective in processing soil slurry, which can be used to strengthen trench excavation walls and prevent dam-side collapse [10]. Sujatsa and Saisree (2019) also reported that using GG in clays with low swelling potential improved consolidation and strength properties [33]. Studies on GG have focused primarily on sand and silt, while its effects on specific soil types, such as swelling clays (BC) and zeolites, remain largely underexplored. Furthermore, the influence of GG’s branched molecular structure and its interactions with soil surface chemistry (e.g., Fe and Mg oxide content) on cohesion has not yet been fully elucidated. In addition to these positive properties, different results have also been reported for biopolymer-soil interactions.

In biopolymer–clay mixtures, mixtures with increased water content absorb water and transfer it to hydrogels. In this case, volumetric expansion (swelling) occurs. As water content increases, the strength and stiffness of the mixtures decrease [15,34]. In this case, it is necessary to discuss the optimal amount of biopolymer in the soil–biopolymer mixture. Çabalar et al. stated that due to the low specific gravity and water absorption property of XG, there was a decrease in MDD values and an increase in OMC values [35]. Similarly, Sujatha and Saisree stated that, in the use of GG, MDD values increased and OMC values decreased, attributing this to the fact that soil treated with GG can be compacted at low energy levels and is not sensitive to changes in water content [33].

In light of this data, the main objective of this study is to systematically investigate the effects of XG and GG biopolymers on three soil types with distinct mineralogical characteristics (bentonite–BC; zeolite silty soil–ZS; red clay–RC) and to elucidate the micro-mechanisms underlying these effects. In this context, the following specific objectives were determined:

(a) To establish a systematic comparison of polymer-mineral interface responses of XG and GG in three distinct soil matrices: BC, ZS, and RC. Unlike previous studies that focus on a single soil type, this approach aims to investigate the dependence of biopolymer performance on soil mineralogy.
(b) To elucidate the micro-causal relationships underlying macro-changes in strength and permeability by directly visualizing the film layers and network structures formed by biopolymer gels on soil surfaces using scanning electron microscopy (SEM).
(c) To test the hypothesis that the molecular structure of biopolymers (linear XG vs. branched GG) plays a decisive role in soil improvement performance, and to identify the most suitable biopolymer type and dosage for each specific soil type.
(d) To fill the research gap regarding the effects of GG on swelling clays and zeolitic soils, by systematically evaluating its interaction with different soil surface chemistries (particularly Fe and Mg oxide content).
(e) To create a database for appropriate engineering applications by determining the most suitable biopolymer and dosage for specific soil types, and to propose optimum application areas (e.g., drainage, sealing barriers, filling) for each soil–biopolymer combination.

In line with the stated objectives, the effects of varying XG and GG doses (0% to 4%) on bentonite (BC), zeolite silty soil (ZS), and red clay (RC) with different mineralogical characteristics were systematically investigated. The basic engineering properties of the mixtures, such as specific gravity, consistency limits, compaction behavior, shear strength parameters, and hydraulic conductivity, were determined experimentally; SEM images were taken of selected mixture samples, and the resulting changes were examined at the micro-scale. Thus, it was shown that the success of biopolymer soil improvement depends critically not only on the type and dosage of biopolymer but also on the mineralogical and chemical characterization of the soil, and optimum application areas (drainage, sealing barrier, filling, etc.) were suggested for each soil–biopolymer combination.

## 2. Materials and Methods

**Figure 1:**
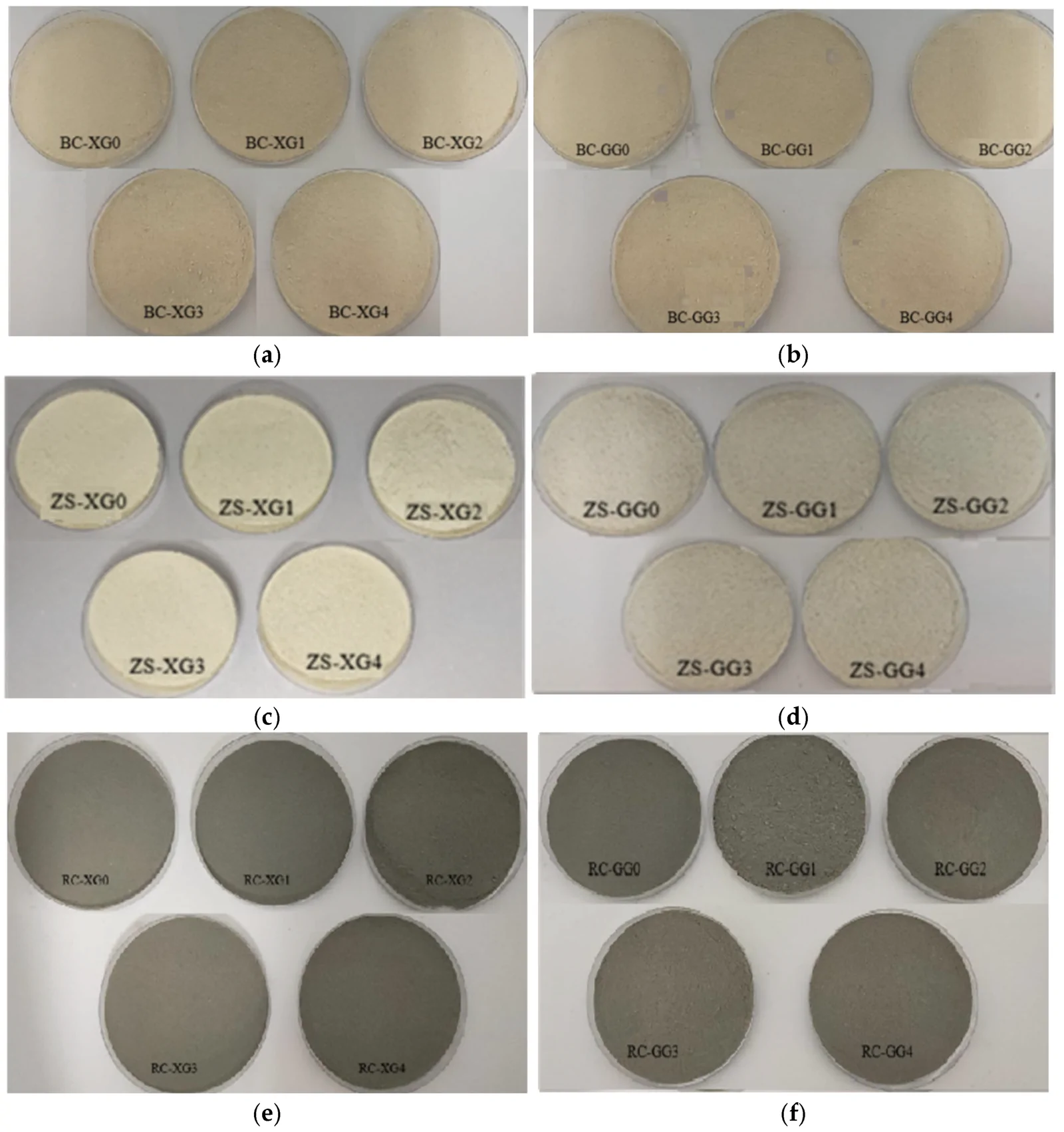
Soil–biopolymer mixture samples used in the study ((**a**). BC-XG; (**b**). BC-GG; (**c**). ZS-XG; (**d**). ZS-GG; (**e**). RC-XG; (**f**). RC-GG samples).

**Figure 2:**
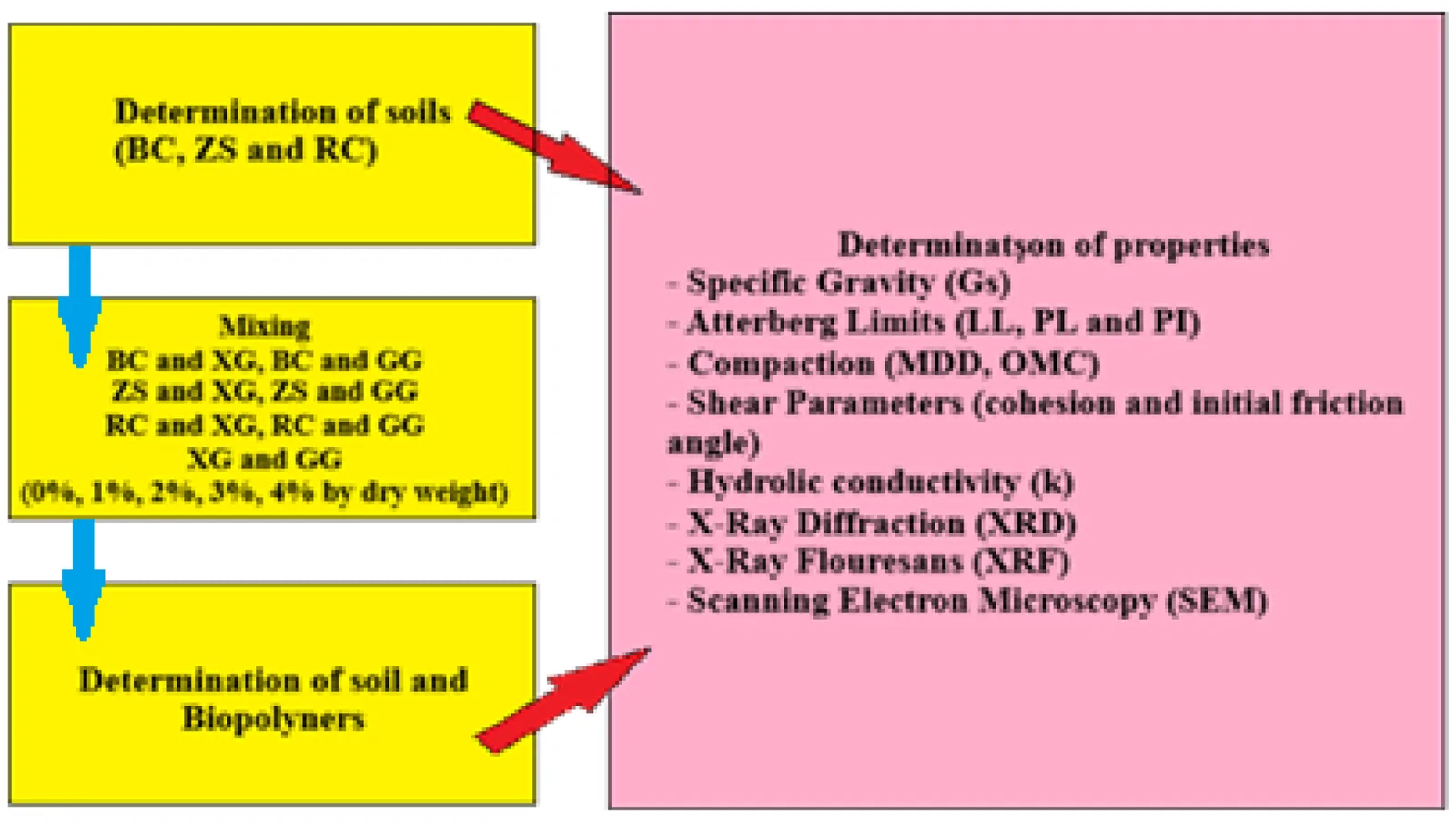
Experimental Methodology of Studies.

In this study, three different types of soil were used in experimental analyses: bentonite (BC), zeolite silty soil (ZS), and red clay (RC). BC samples were obtained from the Battalgazi district of Malatya (Turkey) province, and ZS and RC samples were obtained from the Hekimhan district of Malatya (Turkey) province. The samples were dried at room temperature in the laboratory and ground in a ball mill to obtain fine fractions. In all experiments, the effect of particle size was minimized by passing the dried and ground materials through a 0.075 mm sieve. The biopolymers (XG and GG) used were commercially available and used as received without further purification. All soil–biopolymer mixtures were prepared by dry mixing method. For each mixture, the required amounts of oven-dried soil and biopolymer powder (at ratios of 0%, 1%, 2%, 3%, and 4% by dry weight of the soil) were weighed separately. The components were then mechanically mixed in a laboratory-scale rotary mixer for 10 min at a speed of 60 rpm to ensure complete homogeneity. To prevent segregation and moisture absorption, the mixtures were stored in sealed containers at room temperature for 24 h before testing. The soils and biopolymer mixtures used in the studies are shown in Figure 1; the symbols used are shown in Table 2; and the flowchart of the experiments to be performed on the samples is shown in Figure 2.

### 2.1. Experimental Studies

This section describes in detail the laboratory experiments and test methods used to evaluate the characteristic geotechnical properties of clay soils.

### 2.2. Characterization of the Soils Used

To reveal and characterize the mineralogical and crystallographic differences among the soils used in the studies, XRD, XRF, and SEM analyses were performed on three soil types at the Central Research Laboratory of Kastamonu University (MERLAB-Laboratory Management System-Kastamonu-Turkey) and at İnönü University (IBTAM-Scientific and Technical Research Center-Malatya-Turkey). XRD analyses were carried out using a Rigaku-D/Max 2200 X-ray diffractometer (Rigaku Corporation, Tokyo, Japan) equipped with a graphite monochromator employing Cu radiation with a scan rate of 1 °C/min and a secondary beam graphite monochromator. The spectra were recorded in the 2θ range, XRF analyses using a Xepos II-Spectro (SPECTRO Analytical Instruments GmbH, Kleve, Germany), and SEM image analyses using a Quanta FEG 250-FEI model (FEI Company, Hillsboro, OR, USA) instrument.

### 2.3. Sieve Analysis

Dry sieve analysis was performed on the soils used in the studies according to the ASTM D422 (1998) standard, which is the standard test method to determine the particle size distribution of the soils. Particle size distribution graphs were created using the obtained results [36].

### 2.4. Specific Gravity (Gs)

In accordance with ASTM D854, Gs measurements were repeated three times for each mixture sample. The Gs values of the mixtures were determined by averaging the specific gravity tests performed using a pycnometer [37]. The main purpose of this experiment is to determine the effect of biopolymer addition on the solid phase density of the soil mixture. In the literature, biopolymers, due to their inherently low particle densities (1.4–1.6 g/cm^3^), generally decrease the Gs value when mixed with clay minerals [32,35]. Thus, the magnitude of the dilution effect of low-density biopolymers when mixed with high-density clay minerals can be calculated based on the biopolymer concentration.

### 2.5. Atterberg Limits

Atterberg limit tests were performed in accordance with ASTM D4318 to determine the consistency properties of the samples [38]. Liquid limit (LL) tests were determined using the Casagrande instrument. According to the results of the LL test, which was performed in 4 replicates, the value corresponding to 25 pulses on the graph of pulses versus water content was recorded as the LL value. Plastic limit (PL) tests were determined manually; average values were used after performing four repetitions for each mixture. The plasticity index (PI) was calculated as the difference between LL and PL. The high water-retention and hydrogen-bonding capacity of biopolymers such as XG fundamentally alter soil–water interactions and microstructural bonds [7]. The engineering effects of such biopolymer–clay interactions on soil consistency limits are also documented in the literature [32]. In the literature, the main reasons for the increase in LL and PI values with biopolymer addition are shown as: the increase in the viscosity of the pore water, the three-dimensional network structure formed by the biopolymer gel between the clay particles, and the hydrogen bonds between the clay and the biopolymer [33,38]. This study aimed to determine, specifically through Atterberg limit experiments, how the same biopolymer would behave in different soils.

### 2.6. Standard Compaction Test

According to ASTM D698 [39], the standard Proctor test was performed to determine the compaction properties (optimum water content—OMC, maximum dry density—MDD) of mixture samples. Mixture samples were compacted in Proctor molds using 25 blows per compaction. At this stage, wet bulk weights and average water contents were determined. This process was repeated 5 times, increasing the water content each time. In the dry bulk weight and water content graphs created at the end of the experiment, the peak points were recorded as MDD and OMC values [39]. The low specific gravity and high water-holding capacity of biopolymers such as XG and GG fundamentally alter the compaction behavior of soil–biopolymer mixtures. The literature reports that the addition of biopolymers generally leads to a decrease in MDD and an increase in OMC [33,35]. This is explained by two main mechanisms: the lubricant effect of the viscous gel layer formed between the particles by biopolymers prevents the tight packing of the particles, and the high water-holding capacity of biopolymers necessitates additional water for the soil to reach optimum consistency [21,40].

In this study, OMC and MDD values were determined for each soil–biopolymer combination, and a comparison was conducted. Furthermore, correlations were established between changes in MDD and OMC and changes observed in Atterberg limits (especially PI), and the validity of these mechanisms was tested. The obtained results were then used as a substrate for sample preparation in subsequent shear strength and hydraulic conductivity tests. Thus, the performance of different biopolymer dosages was made comparable under the same compression conditions.

### 2.7. Shear Strength Test

Shear strength tests of soil and mixture samples were performed according to ASTM D3080 standard using a shear box test apparatus [41]. The tests were conducted at the Academy Soil Investigation Laboratory (Ankara, Turkey). A fully automatic shear box device (UTS-2060, UTS Test Systems, Rock Hill, SC, USA) with a maximum shear capacity of 5 kN and a constant shear rate of 1 mm/min was used in the tests. The samples were prepared at the optimum moisture content (OMC) determined by previous compaction tests and molded to have a cross-sectional area of 6 cm × 6 cm and a thickness of 2 cm. Shear box tests were performed under three different vertical stresses of 50, 100, and 200 kPa for each mixture sample, and the shear strength parameters (cohesion-c, internal friction angle-Ø) were determined.

The literature reports that biopolymers generally increase the shear strength of soils [7,12,21]. This study aimed to test the extent to which this general acceptance holds across different types of soil and across different types and ratios of biopolymers.

### 2.8. Hydraulic Conductivity Test

Permeability (hydraulic conductivity) tests were performed using the falling-head permeability test in accordance with ASTM D5856-95 [42]. This method was chosen to precisely measure the hydraulic conductivity of soil and biopolymer-soil mixtures with low permeability. The tests were performed on samples prepared with OMCs determined by compaction tests, and average values were used after performing three replicates for each mixture. The permeability tests were conducted at the Academy Soil Survey Laboratory (Ankara, Turkey) using a rigid-wall compaction-mold permeameter (Model 57-3700, ELE International, Leighton Buzzard, UK). The aim of the permeability tests performed on soil and mixture samples was to determine the effect of XG and GG on the hydraulic conductivity of three different soils. Thus, by evaluating the obtained k values together with the shear strength parameters (c, Ø) determined from the same samples, the suitability of each soil–biopolymer combination for special applications, such as sealing barriers, can be discussed.

## 3. Results and Discussion

### 3.1. Results of Characterization of the Soils Used

**Figure 3:**
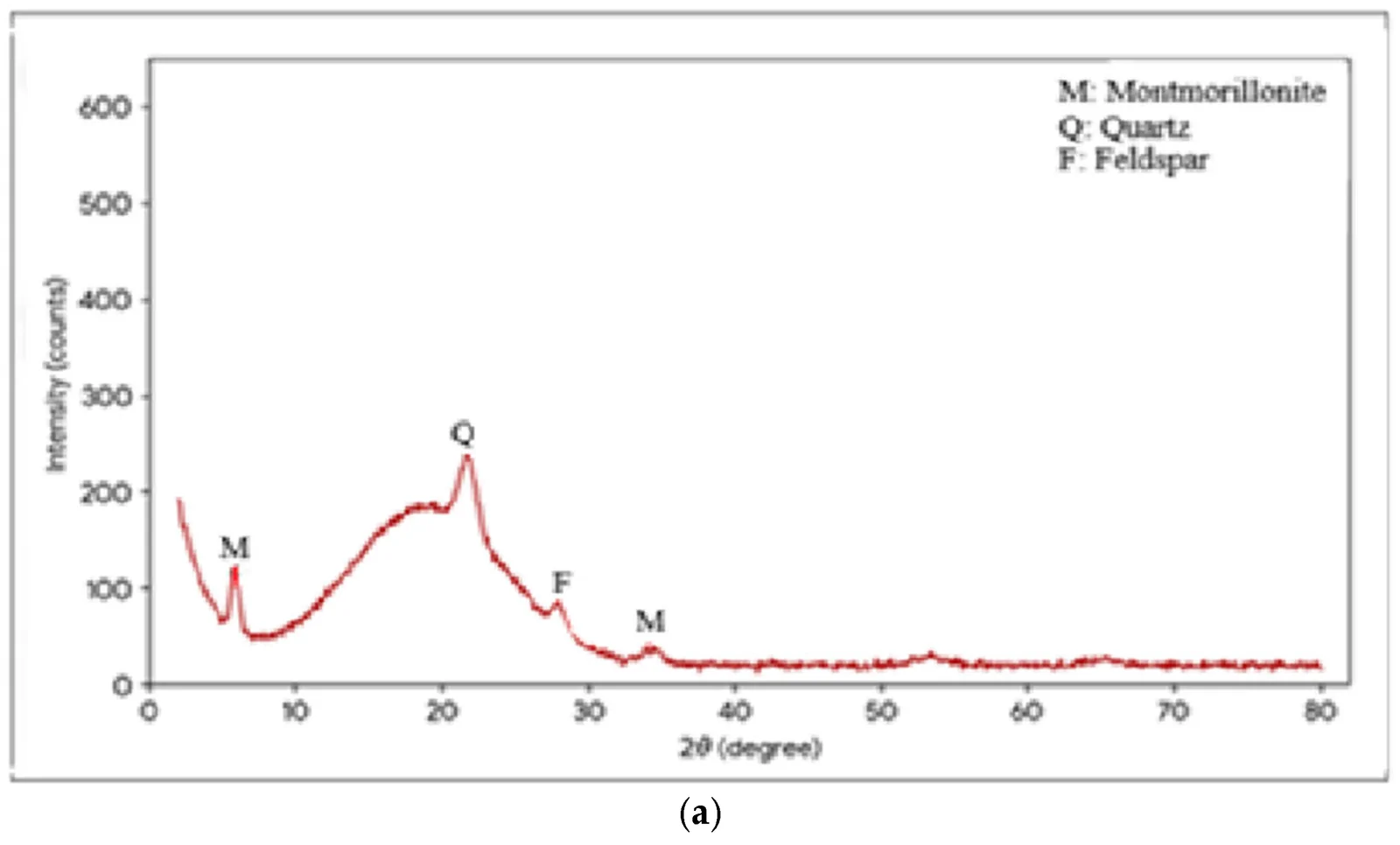

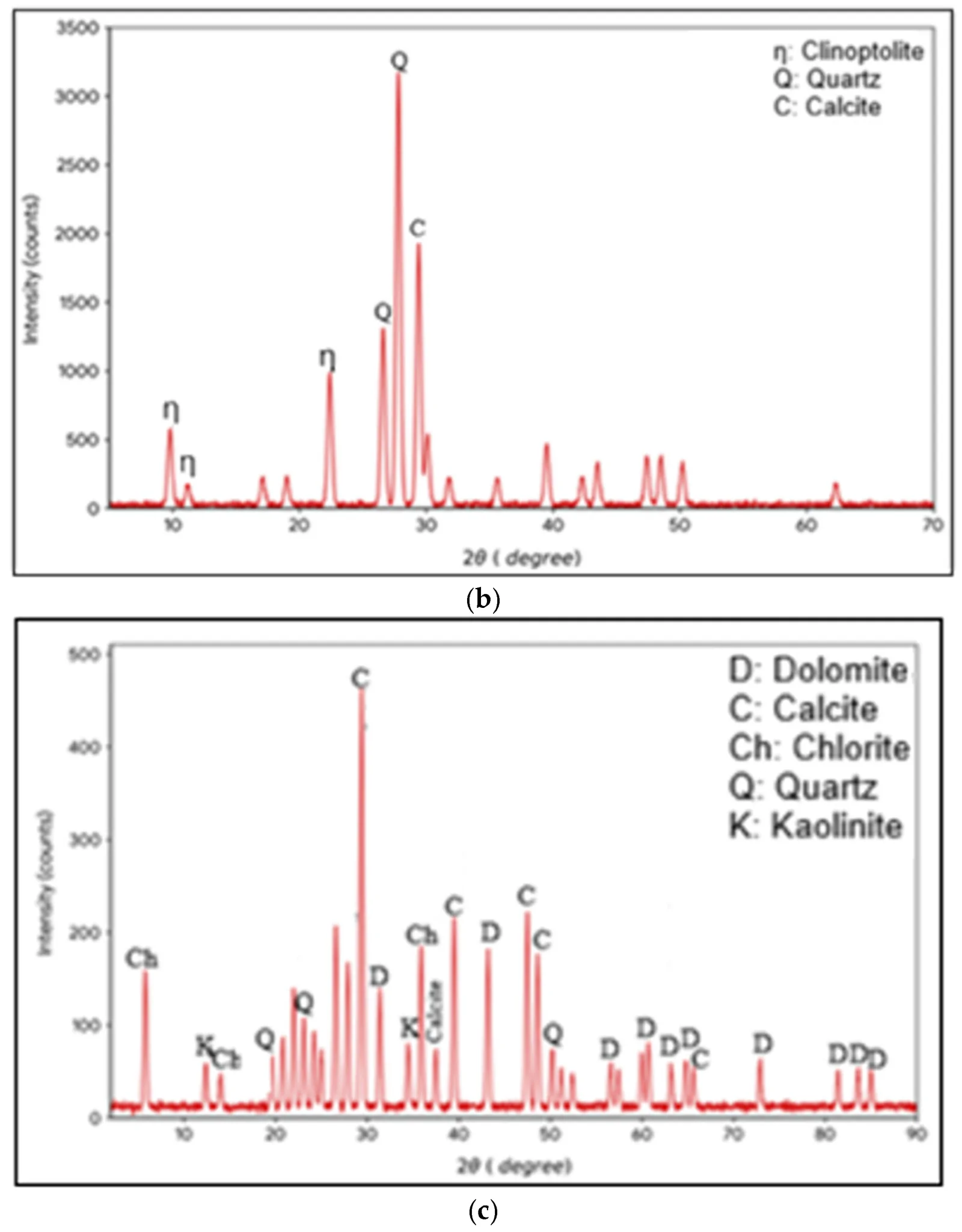
XRD analysis results of soils ((**a**). BC; (**b**). ZS; (**c**). RC).

**Figure 4:**
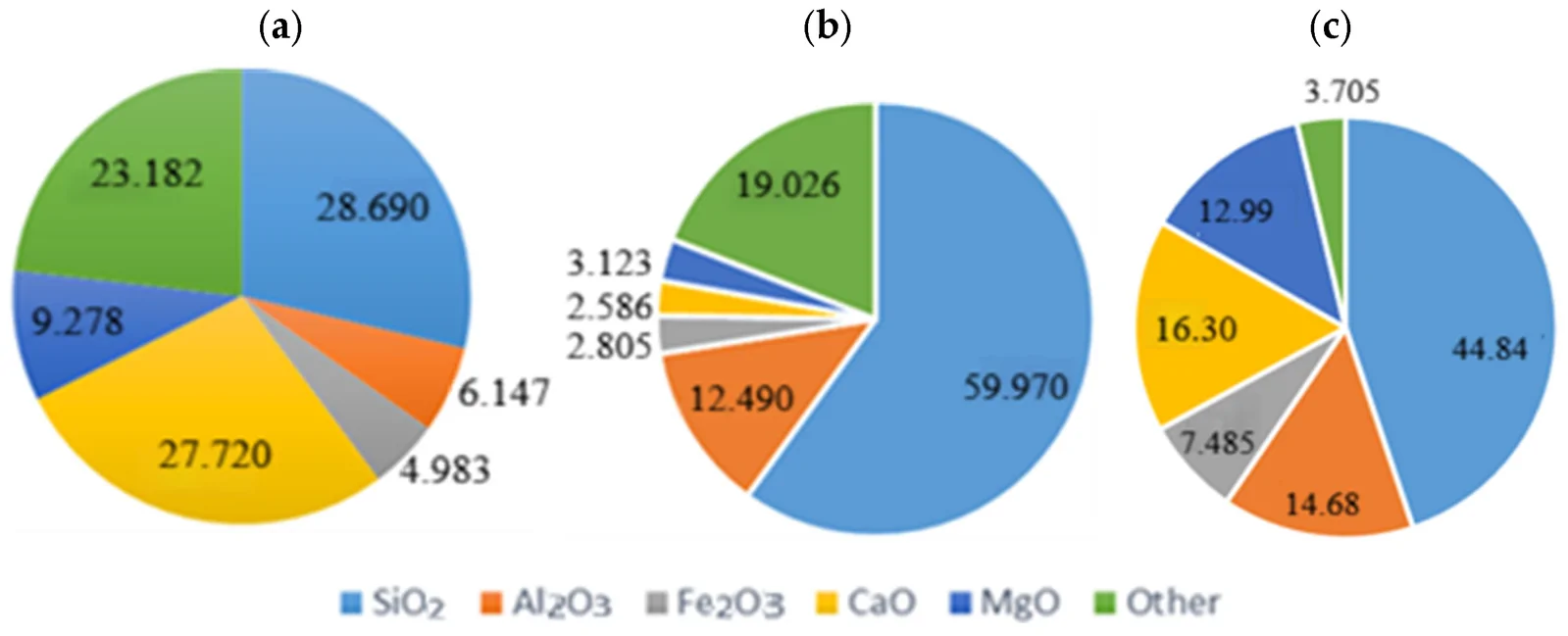
XRF analysis results of soil minerals ((**a**). BC, (**b**). ZS, (**c**). RC).

**Figure 5:**
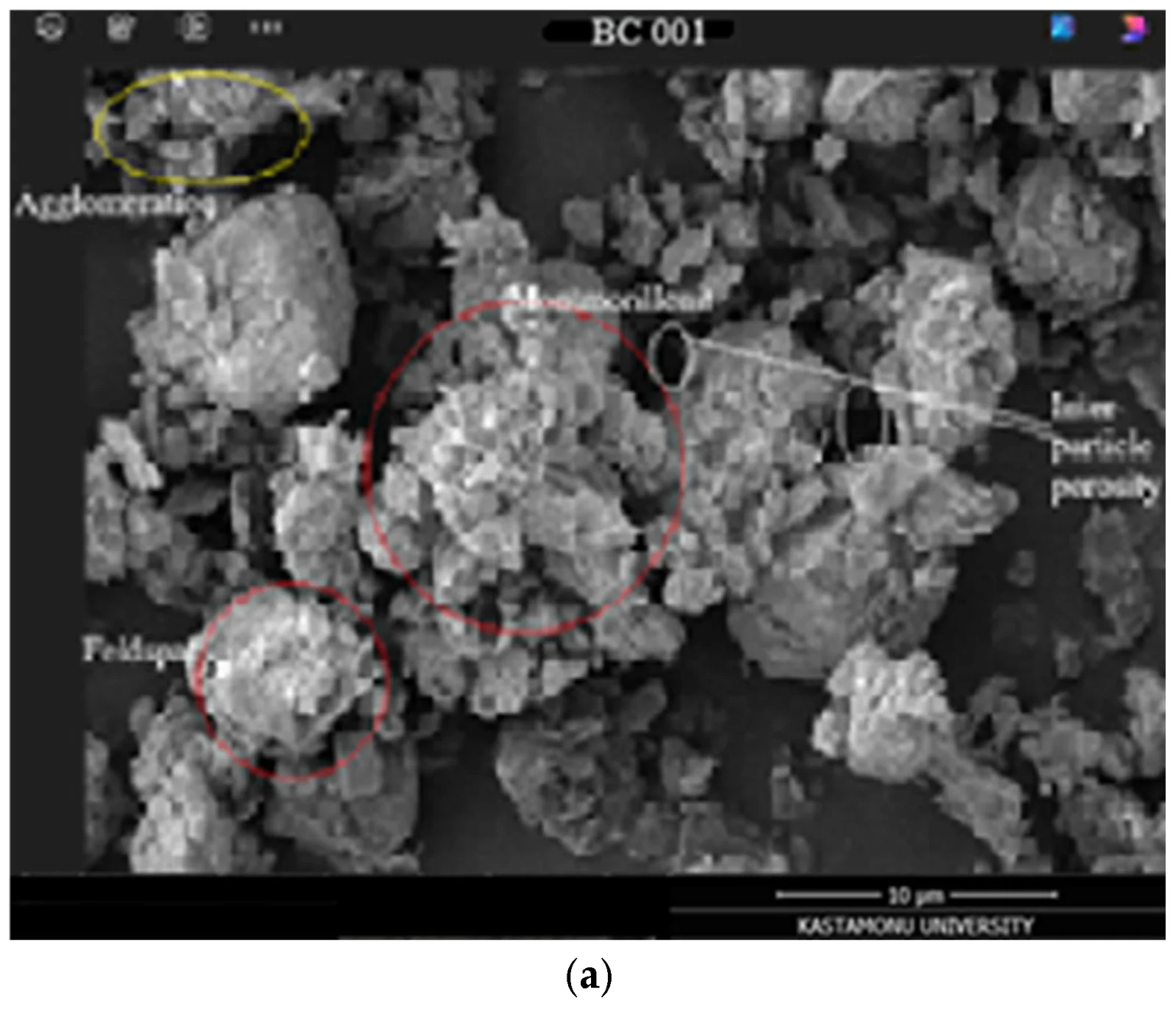

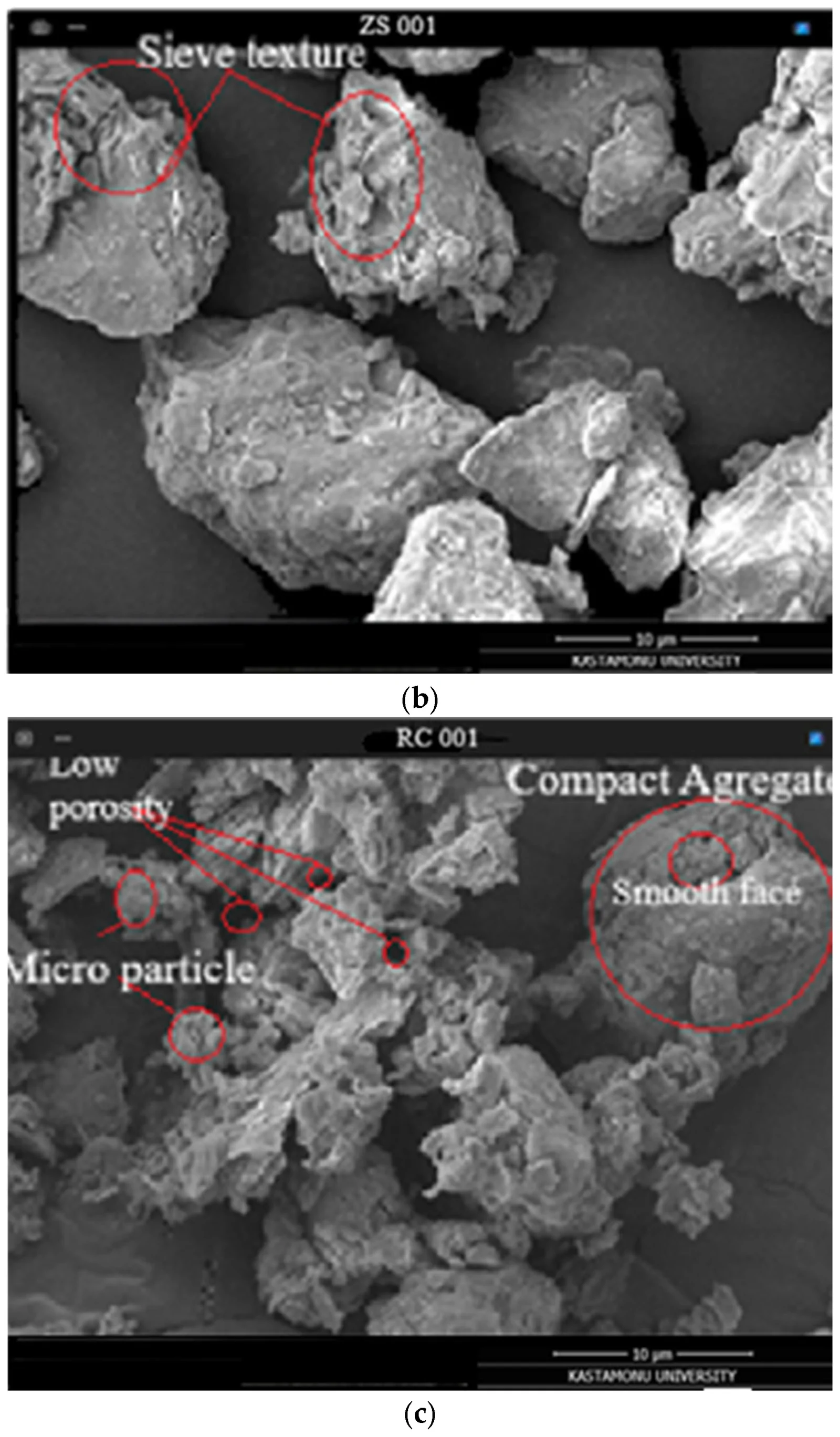
SEM images of soils ((**a**). BC, (**b**). ZS, (**c**). RC).

The XRD results of the soils used in the studies are given in Figure 3, the XRF results in Figure 4, and the SEM image analysis results in Figure 5.

Mineralogical characterization of the BC sample was conducted using X-ray diffraction (XRD) analysis (Figure 3a). The XRD pattern revealed the characteristic (001) basal reflection of the smectite group mineral montmorillonite at approximately 5.8° 2θ. This peak was assigned to the Ca-montmorillonite phase by comparison with the International Center for Diffraction Data (ICDD) Powder Diffraction File (PDF) card number 00-003-0015. The identification is further corroborated by the elevated CaO content detected in the X-ray fluorescence (XRF) analysis. The broad amorphous region between 10° and 35° 2θ suggests the presence of dense silica or glassy phases within the matrix. Additionally, crystalline peaks observed in the 22° to 28° 2θ range were attributed to the feldspar phase. Feldspar is recognized as a secondary mineral commonly present in bentonite deposits [43].

The X-ray diffraction (XRD) pattern of the ZS sample (Figure 3b) demonstrates a multiphase structure, with clinoptilolite identified as the primary mineral phase. This identification is substantiated by reflections at 9.9°, 11.2°, and 22.4° 2θ, which are characteristic low-angle peaks of clinoptilolite. Additional secondary peaks at 2θ = 13.0°, 17.3°, and 19.1° further corroborate this assignment [44]. The pattern also reveals peaks corresponding to quartz impurity phases at 20.8°, 26.6°, and 27.8° 2θ, as well as a calcite impurity phase at 29.4° 2θ [45]. The occurrence of secondary minerals such as quartz and calcite in natural clinoptilolite samples is frequently reported in the literature. X-ray fluorescence (XRF) analyses further support these findings. The high SiO_2_ content (59.97%), low CaO content (2.59%), and Si/Al ratio by weight (4.80) align with the typical range (4.5–6.0) reported for clinoptilolite [46]. Therefore, the main phase of the ZS sample is clinoptilolite, accompanied by quartz and calcite impurities (ICDD PDF #01-0646; ICDD PDF #01-0832).

Mineralogical characterization of the RC sample was conducted using X-ray diffraction (XRD) analysis (Figure 3c). The resulting XRD pattern was compared with the International Center for Diffraction Data (ICDD) database and indicates a heterogeneous phase distribution, primarily comprising calcite (ICDD PDF card no 05-0586), dolomite (ICDD PDF card no 36-0426), quartz (ICDD PDF card no 46-1045), and clay minerals including kaolinite, chlorite, and illite.

The highest intensity peak at 29.4° 2θ corresponds to the calcite phase, as further supported by the elevated CaO content (16.30%) measured by XRF. A prominent secondary peak at 30.9° 2θ indicates the presence of dolomite, corroborated by the high MgO content (12.99%) in the XRF results. The simultaneous occurrence of carbonate minerals such as calcite and dolomite is characteristic of clays formed in carbonate-rich depositional environments. Analysis of the clay fraction peaks identified kaolinite (12.4° 2θ), chlorite (6.2° 2θ), and illite (8.8° 2θ) phases. Quartz was identified by its characteristic peaks at 2θ = 20.8° and 26.6°. This mineral assemblage indicates that the RC sample is a dolomitic-limestone residual clay formed under weathering conditions. The Al_2_O_3_ (14.68%) and SiO_2_ (44.84%) concentrations detected in the XRF analysis further support the predominance of kaolinitic clays. Comparable mineralogical compositions, including quartz, calcite, dolomite, kaolinite, illite, and chlorite, are frequently reported in red clays and Neogene clays globally [47].

The major oxide compositions of the soil minerals were determined using XRF, and the results are shown in Figure 4. The results show that the soils differ significantly from one another. The XRF results show that BC is a calcium–magnesium smectite group clay mineral and contains a significant amount of free carbonate. The Si/Al ratio is 4.67 by weight.

ZS contains a high SiO_2_ content (59.97%) and a moderate Al_2_O_3_ content (12.49%). The Si/Al ratio by weight is 4.80. This value remains within the typical range (4.5–6.0) reported in the literature for clinoptilolite [48]. The low CaO content (2.59%) suggests that the exchangeable cations are predominantly Na^+^ and K^+^ rather than Ca^2+^. MgO (3.12%) and Fe_2_O_3_ (2.81%) are at low levels, indicating that the sample is a near-pure clinoptilolite and does not contain significant smectite admixture.

XRF results for the RC indicate that the sample has a carbonate-silica mass characteristic, consisting predominantly of silica and calcium. The highest value in the structure is 44.84%, corresponding to the SiO_2_ phase and directly indicating the presence of crystalline quartz in the XRD pattern. The second major component is CaO, with a high lime content of 16.30%, chemically confirming the calcite phase, which is the most intense peak in the XRD pattern. The Al_2_O_3_ content, which forms the basis of the aluminosilicate matrix, was determined to be 14.68%. The Fe_2_O_3_ content, which gives the sample its characteristic reddish-brown color and accounts for a significant share of the structure, is 12.99%. The remaining minor segments of 7.48% and 3.70% represent alkali metal oxides MgO, Na_2_O, K_2_O, and trace elements.

SEM images of BC, ZS, and RC samples reveal significant morphological differences directly related to their mineralogical composition (XRF results) and engineering behavior (index properties).

SEM analysis of BC reveals a heterogeneous, porous, and agglomerated matrix structure. In general, the morphological structure shows thin, wavy, and twisted plate-like structures that come together to form larger aggregates. This layered and twisted microstructure is consistent with the typical “cornflake-like” morphology of the montmorillonite phase of smectite-group clay minerals, as supported by XRD data (Figure 5a).

In contrast to the main plate-like structures, some primary grains with angular and block-like morphology are also noticeable in the clay matrix. In addition, the prominent micro-pores (intergranular porosity) and high surface roughness between the aggregates confirm the open pore network and enhanced specific surface area of the material. The porous and irregular microstructure underlies the engineering properties of bentonite, such as high plasticity and low hydraulic conductivity [49].

SEM images of ZS clearly show a regular sieve texture characteristic of clinoptilolite, extending vertically and in parallel within the central crystalline blocks. Around this crystalline structure, irregular clusters with rough, curved surfaces are evident. The surfaces of the grains are quite rough and indented. This indicates that the material has a high specific surface area (Figure 5b).

SEM analysis of RC shows that it has a significantly more massive, denser, and more compact morphology than other soil samples. In the SEM image, large, hard, block-like aggregates (clustered structures) predominate over individual leaf-like layers. The high silica (SiO_2_, 44.84%) and calcium (CaO, 16.30%) contents detected in the XRF data suggest that these large blocks may be tightly stacked structures composed of hard crystalline minerals such as quartz and calcite-feldspar (Figure 5c).

Smaller, irregularly shaped micro-particles are observed adhering to the surfaces and surroundings of the larger aggregates. These small formations and rough structures within the matrix may represent iron oxide minerals (hematite, goethite, etc.) formed by the iron element (Fe_2_O_3_, 7.485%), which is prominently present in Figure 4. On the other hand, due to the bulky and compact stacking structure, the intergranular porosity level appears relatively low. The RC used in the studies was taken from clays used in brick making in the Hekimhan (Malatya, Turkey) region.

The SEM image of the RC generally shows overlapping, foliated (scaly/layered) structures with irregular boundaries (Figure 5c). The large mass clearly visible on the right is a dense, tight agglomerate formed by the aggregation of very small submicron particles. It exhibits a more clustered appearance compared to zeolite. Micron-sized voids exist between the layers. The RC sample exhibits a more amorphous, foliate/clayey, and densely clustered microstructure compared to the angular and more distinct crystalline structure of ZS (zeolite silty soil).

### 3.2. Geotechnical Properties of Soils

Table 3 presents the basic geotechnical properties of three different soil minerals. According to the Unified Soil Classification (USCS), BCZS and RC are defined as MH (High Plasticity Inorganic Silt) and CL (Low/Medium Plasticity Inorganic Clay), respectively [50]. Although zeolite is not a clay mineralogically, due to its grain size and geotechnical plasticity properties (PI: 40.76%), it exhibits fine-grained clayey soil (CH; high plasticity inorganic clay) behavior in the Unified Soil Classification System (USCS).

Specific gravity (Gs) values were obtained as 2.447 in BC, 2.550 in RC, and 1.740 in ZS. The low specific gravity of ZS is due to the characteristic high void ratio of zeolites. The higher Gs value of RC compared to others is consistent with the silica, iron, and magnesium oxides it contains (44.84% SiO_2_, 7.485% Fe_2_O_3_, and 12.99% MgO, respectively, in XRF analysis).

The highest MDD value was observed in RC (1.795 g/cm^3^), followed by BC (1.360 g/cm^3^) and ZS (1.310 g/cm^3^). OMC values were lowest in RC (15.00%), while BC (27.00%) and ZC (28.50%) exhibited higher OMC values. The high MDD and low OMC values of RC indicate that it has a less plastic, more densely packed structure.

According to shear strength parameters, the highest cohesion (c) value was measured in ZS (66.30 kPa), followed by RC (58.00 kPa) and BC (56.30 kPa). Internal friction angle (Ø) values reached their highest level in BC (49.87°), followed by ZS (31.37°) and RC (25.02°).

Based on the hydraulic conductivity (k) values, the lowest flow velocity was observed in ZS (0.107 × 10^−9^ m/s), followed by RC (4.34 × 10^−9^ m/s) and BC (7.56 × 10^−9^ m/s). All samples are classified as “low permeability materials” from a geotechnical perspective. The fact that ZS has the lowest hydraulic conductivity is related to its high plasticity (PI: 40.76%) in the CH class and to zeolitic channels that trap water molecules through strong crystal bonds.

### 3.3. Microstructure of Mixture (SEM Analysis of Mixture)

**Figure 6:**
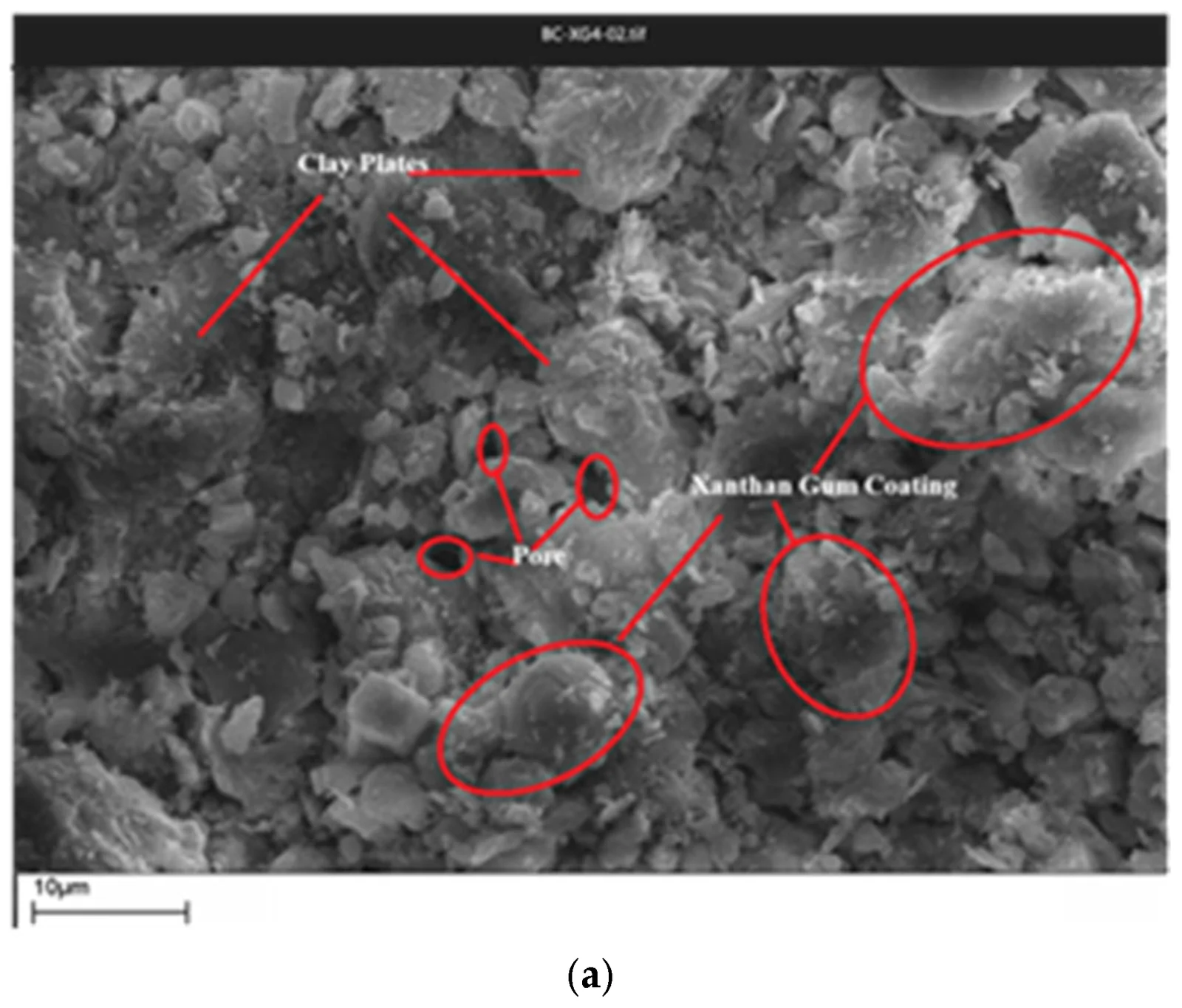

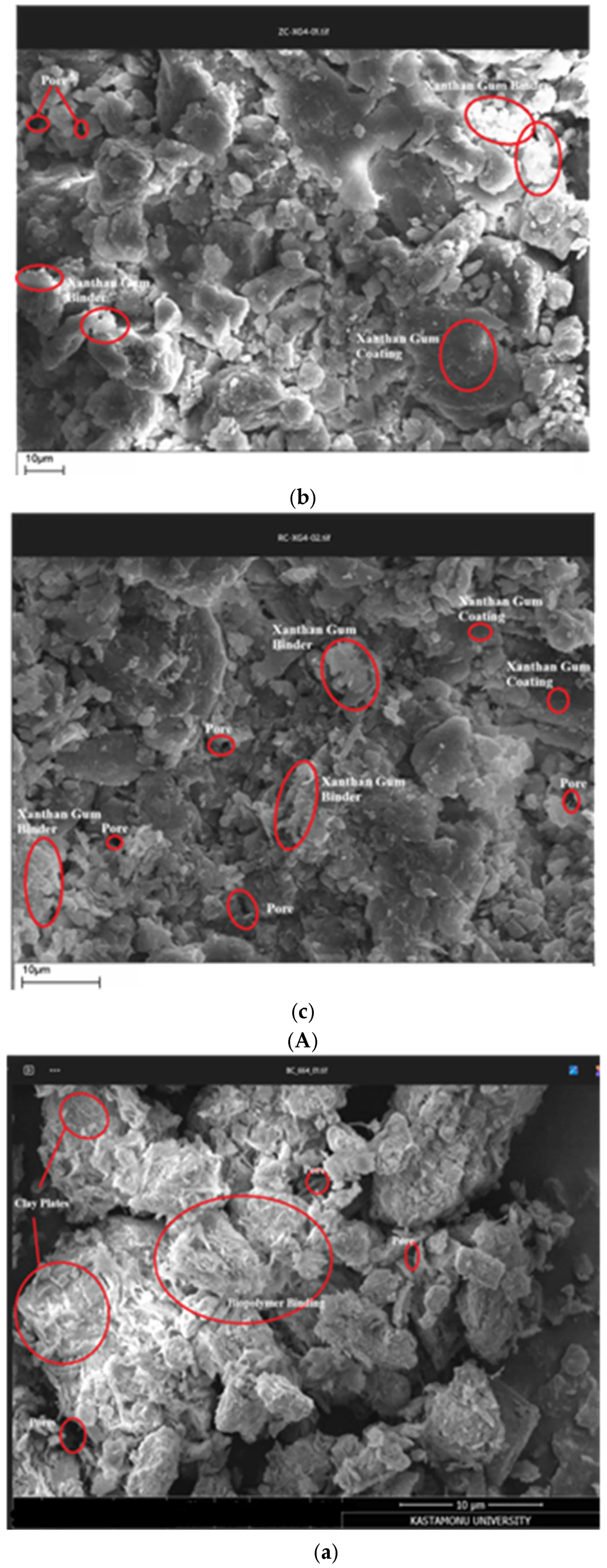

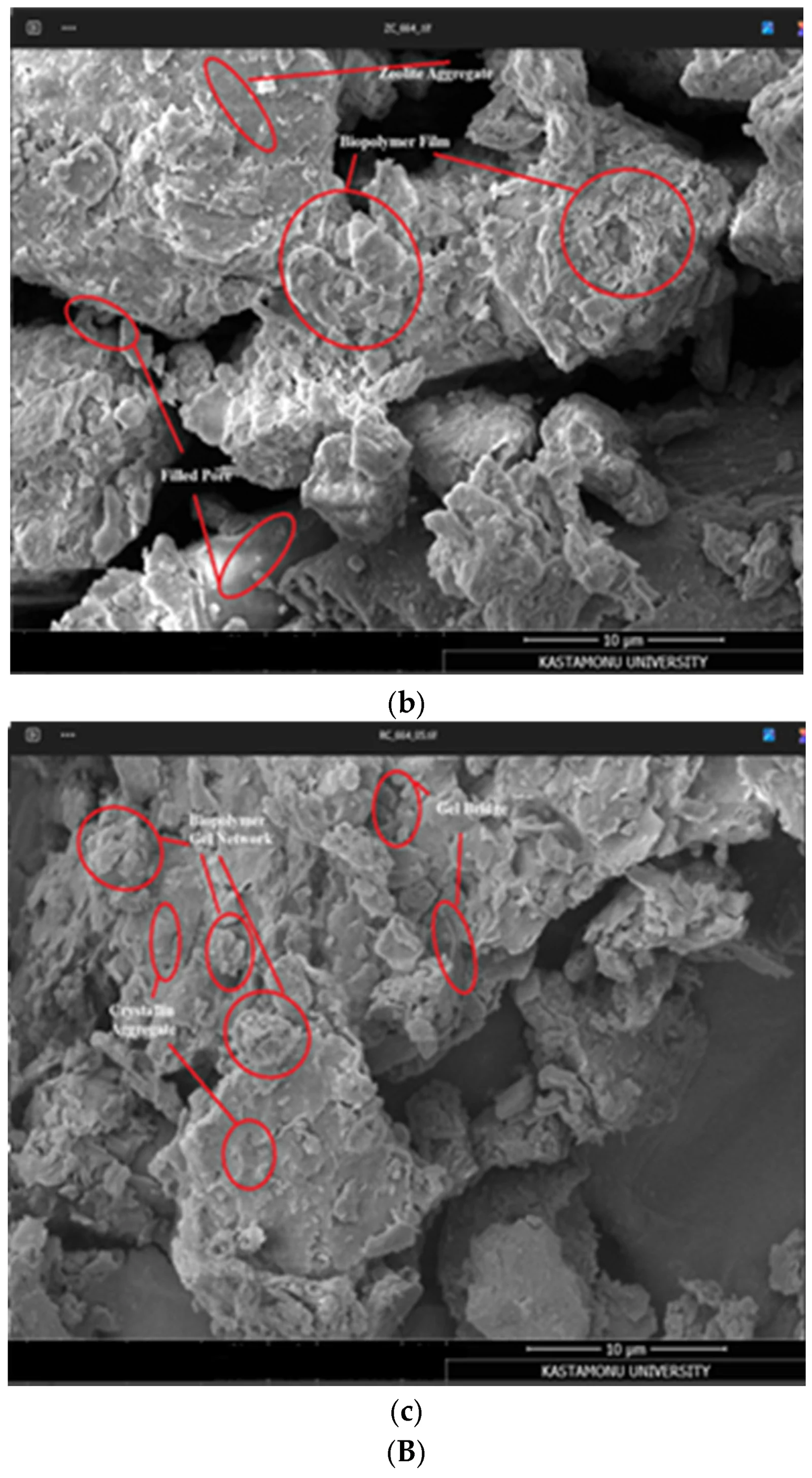
(**A**) SEM images of mixture samples with 4% XG ((**a**). BC-XG4, (**b**). ZS-XG4, (**c**). RC-XG4). (**B**) SEM images of mixture samples with 4% GG additives ((**a**). BC-GG4, (**b**). ZS-GG4, (**c**). RC-GG4).

SEM analysis results for the mixture samples are given in Figure 6A (for 4% XG) and Figure 6B (for 4% GG).

#### 3.3.1. Microstructure of BC Mixtures

The SEM image of the BC-XG4 sample shows that the addition of XG transforms the loose and disordered aggregate structure of pure BC (Figure 5a and Figure 6A) into a compact and integrated microstructure. The film-like polymeric structures developing between the clay plates demonstrate that XG enhances the aggregation mechanism by coating the particle surfaces. The significant reduction in interparticle void volume is concrete evidence of the biopolymer’s pore-filling effect.

The SEM image of the BC-GG4 sample is given in Figure 6B. The image shows that the addition of GG develops a biopolymer-derived network structure between the clay particles. The connections between the clay plates are characterized by fibrous and bridge-like formations, indicating increased interparticle interaction. The network structure observed in the image reveals that GG binds the clay particles together, creating more stable aggregates. Although a denser microstructure has developed compared with the pure BC sample, some micropores are still present. This suggests that GG’s fundamental stabilization mechanism is related to the formation of binding bridges between particles rather than gap filling.

#### 3.3.2. Microstructure of ZS Mixtures

The SEM image of the ZS-XG4 sample shows significant microstructural changes resulting from the addition of XG (Figure 6A). The open-void and loose aggregate structure observed in the pure ZS sample (Figure 5b) has been replaced by a more compact and integrated microstructure. The continuous biopolymer films forming on particle surfaces and the binding regions formed between particles indicate that XG provides stabilization through both coating and bridging mechanisms. Furthermore, the decrease in pore volume and the denser packing of aggregates indicate the biopolymer’s void-filling effect.

The SEM image of the ZS-GG4 sample shows that the addition of GG led to significant aggregation and bridging mechanisms between the zeolite particles (Figure 6B). As a result of the addition of biopolymers, the contact areas between the particles increased, and more stable aggregate structures formed. Film-like biopolymer phases and network structures connecting the particles are observed within the microstructure. Although there is a decrease in void volume compared to the pure ZS sample, some micropores are preserved. This indicates that the primary effect of GG is to enhance interparticle binding rather than filling gaps.

#### 3.3.3. Microstructure of RC Mixtures

The SEM image of the RC-XG4 sample is given in Figure 6A. It is observed that the XG additive creates strong bonding regions between the particles. Continuous film-like coatings have formed on the particle surfaces after the addition of the biopolymer. Furthermore, a significant reduction in the voids between the aggregates is observed. The polymeric phases formed by XG bind the particles together, resulting in a more integrated and denser microstructure. The loose structure observed in the pure RC sample (Figure 5c) has largely disappeared, resulting in a more homogeneous texture.

The SEM image of the RC-GG4 sample shows that the GG additive creates enhanced biopolymer networks and bridging structures between the particles (Figure 6B). The gel-like phases observed in the image have increased the degree of aggregation by establishing physical connections between the particles. Unlike the XG-added sample, GG’s dominant effect appears to be the formation of three-dimensional network structures that bind the particles together. These network structures restrict particle movement and ensure the formation of more stable aggregates. As a result, the microstructure has become more integrated, and interparticle bonding has significantly increased. These mineralogical (XRD), chemical (XRF), and morphological (SEM) characterizations formed the basis for understanding the engineering behavior of pure clays (Table 3) and relating the macro-scale changes observed after biopolymer addition to microstructural mechanisms.

### 3.4. Index and Compaction Properties of Mixtures

Experimental results show that adding XG and GG biopolymers at different ratios (0%, 1%, 2%, 3%, 4%) fundamentally alters the physical and compaction behavior of the three selected soils (BC, ZS, RC). The results indicate that the degree of these changes depends not only on the type and ratio of biopolymer but also on the mineralogy of the soil used. The index and compaction properties of the mixtures are given in Table 4.

#### 3.4.1. Results of Specific Gravity (Gs) Tests

**Figure 7:**
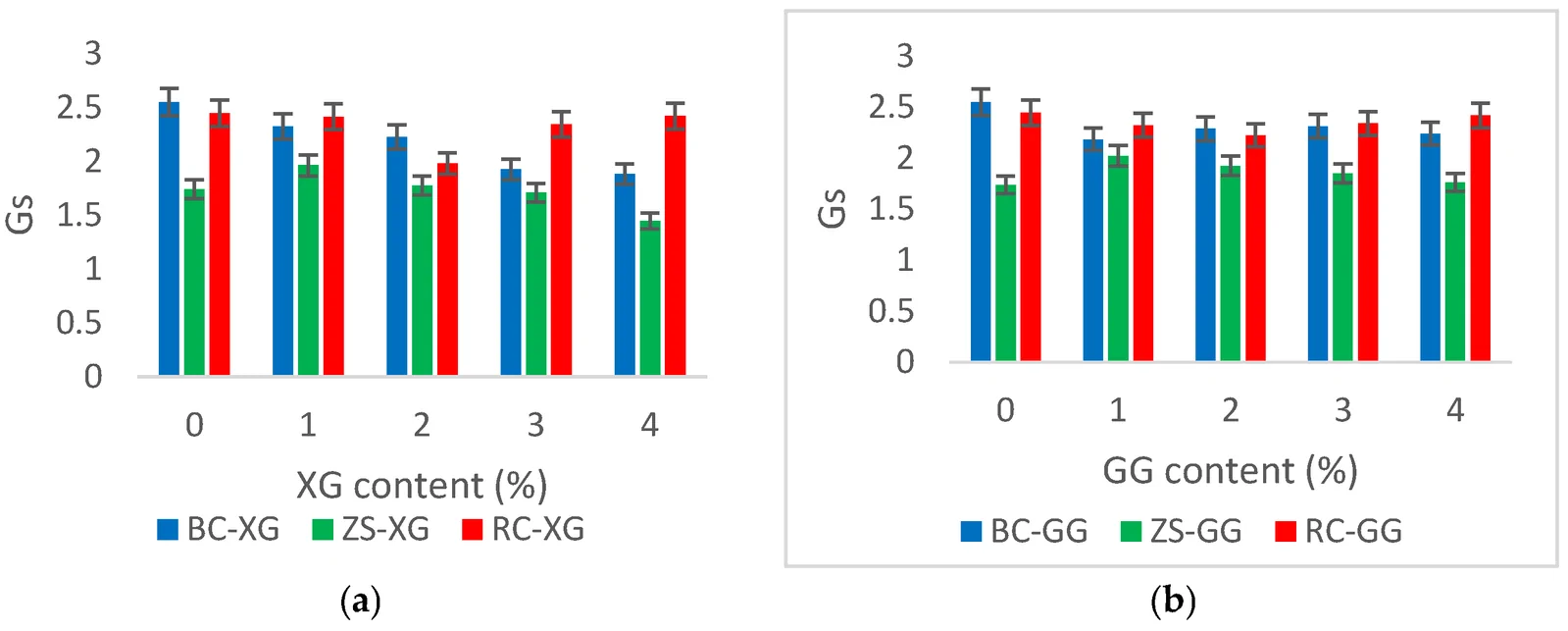
Variation in Gs with (**a**). XG; (**b**). GG content in different soil–biopolymer mixtures.

Changes in specific gravity (Gs) values are an important indicator of the physical and chemical effects of biopolymers in soil mixtures. The Gs test results for the mixture samples are given in Figure 7.

The initial Gs value of BC is 2.447. However, with the addition of XG and GG polymers to the mixture, Gs values decreased with increasing biopolymer amount. In the BC-XG series, the Gs value decreased from 2.447 in the reference sample (BC-XG0) to 1.883 at a 4% dosage (BC-XG4), showing a decrease of approximately 23.05%. Using GG, this decrease was more stable, with a value of 2.186 in the BC-GG4 sample, indicating a decrease of approximately 14%. This decrease in Gs values can be explained by the fact that biopolymers have a relatively low density compared to soil minerals, creating a volumetric “dilution” effect in the soil structure. In contrast, more complex mechanisms were observed in ZS and RC samples depending on the biopolymer type and dosage. In the ZS series, at low dosages of both XG and GG additives (1% and 2%), it was found that the Gs values increased significantly compared to the reference sample (1.740) (ZS-XG1: 1.962, ZS-XG2: 1.775, and ZS-GG1: 2.023, ZS-GG2: 1.924) (Figure 7). This can be explained by the fact that the porous ZS structure, which typically has a high internal void ratio, is filled with low doses of biopolymer gels, thereby increasing the density of the solid phase. The fact that the ZS-GG4 sample (1.764) remained above the reference value despite the onset of dilution effect at high dosages confirms this mechanism. When the SEM image of the ZS-GG4 sample given in Figure 6B is examined, it is seen that the zeolite aggregates are completely surrounded by the GG gel, the intercrystalline voids (filled pores) are filled, and a biopolymer film is formed. This microstructural filling and interlocking mechanism balances the light pore structure of the zeolite, maintaining the density of the solid phase.

In calcite- and quartz-rich RC, both XG and GG usage showed similar trends with dosage. Gs values initially decreased, reaching minimum levels in RC-XG2 (1.979) and RC-GG2 (2.226) samples, respectively, but increased again at 3% and 4% dosages, approaching the reference value (RC-XG4: 2.419, RC-GG4: 2.419). This unique behavior confirms that the biopolymer–clay interaction mechanism reverses beyond a certain threshold dosage, as reported in the literature; at high biopolymer concentrations, the biopolymer chains form tight networks, re-densifying the solid-phase structure [51].

The densification observed in the RC sample with 4% GG (RC-GG4) content is also clearly confirmed at the microstructural level. When the SEM image of the RC-GG4 sample is examined in Figure 6B, it is seen that the high concentration of biopolymer gel surrounds the hard crystalline aggregates (block-like structures) in the mixture with a strong network-like structure (matrix-spanning network). The sharply defined bulk separations in the pure soil structure have been replaced by a larger, more interlocked structure due to the high GG chain dose in the mixture. These gel bridges and the coating layers formed between the particles tightly hold the mineral blocks together, reducing structural voids and providing fundamental physical evidence that the density of the mixture has been increased back to reference levels (Gs).

#### 3.4.2. Results of Atterberg Limits Tests

**Figure 8:**
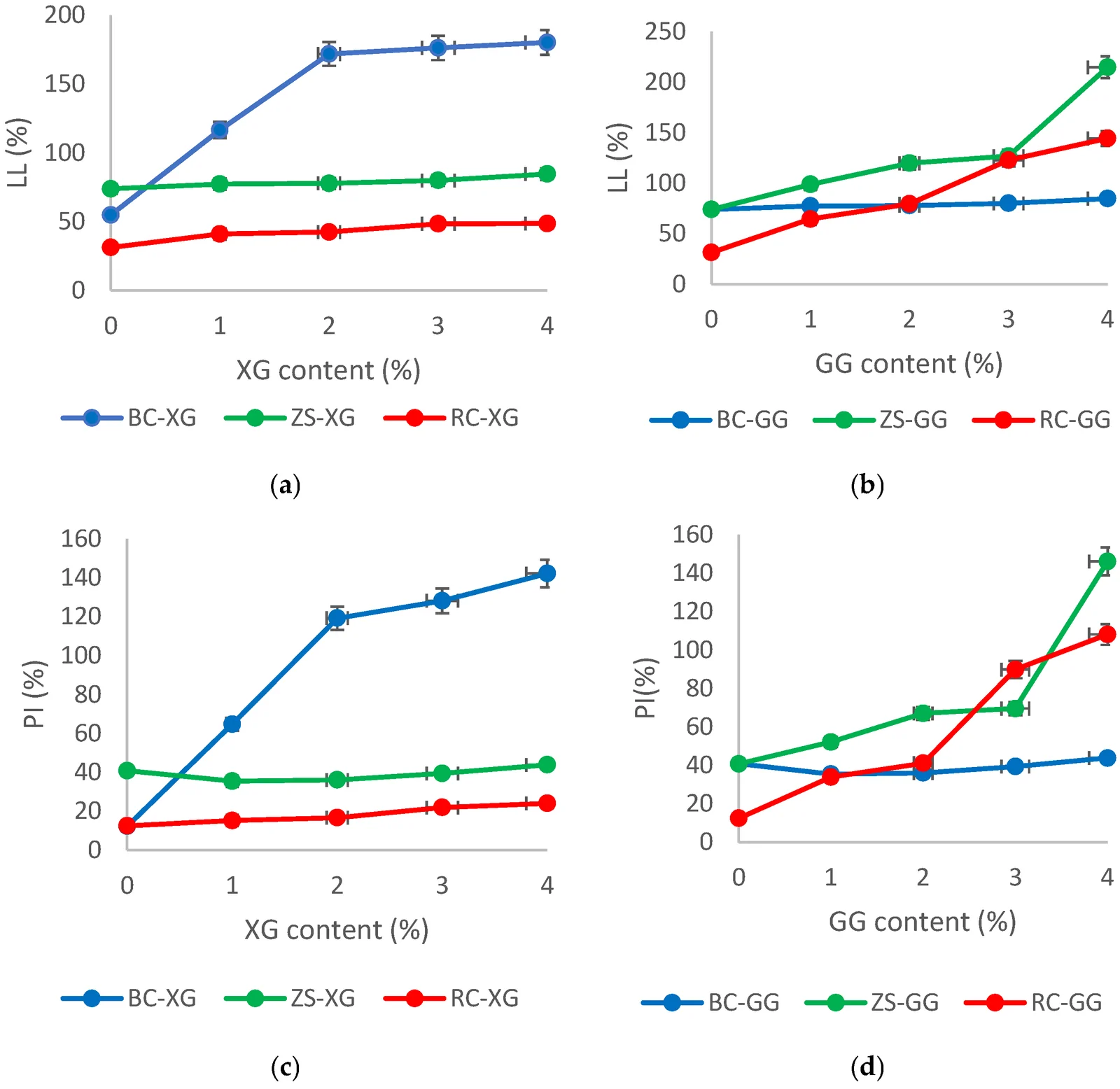
Variation in Atterberg Limits with biopolymer content: (**a**). LL for XG mixtures, (**b**). LL for GG mixtures, (**c**). PI for XG mixtures, (**d**). PI for GG mixtures.

The effects of biopolymer use on Atterberg Limits are the most striking evidence of the hydrophilic nature of biopolymers. The LL and PI test results for the mixture samples are given in Figure 8.

The effect of XG use on LL and PI values in the mixtures varied with soil type (Figure 8a–c). In BC mixtures, an increase in LL values was observed with increasing XG. At a dosage of 4% XG (XG4), compared to the reference samples (XG0), an increase of 228.5% in LL values was obtained in BC mixtures, 14.6% in ZS mixtures, and 55.5% in RC mixtures. In PI values, an increase of 1060% was obtained in BC mixtures, 7.5% in ZS mixtures, and 93% in RC mixtures. The high BC yield relative to others can be explained by the high swelling potential and ion-exchange capacity of BC, which interact more strongly with XG. The main reason for the high increases in PI values is related to the fact that PL values remain constant or change at very low rates despite the sharp increase in LL (PI = LL − PL). When the water content decreases, the bond between the biopolymer and the soil remains strong, making it difficult for the sample to solidify, thus resulting in low PL values. This shows that biopolymer-soil bonds retain their strength even as water content decreases, and that the soil has difficulty solidifying [32]. It can be said that the type of soil is more effective than the biopolymer content and amount in mixtures made with XG.

GG addition caused continuous and extraordinary increases in LL and PI values in all soil types (Figure 8b–d). These increases reached their highest values at a GG dosage of 4% (GG4). For BC, LL increased by 212% and PI by 813%, for ZS, LL increased by 191% and PI by 258%, and for RC, LL increased by 361% and PI by 770%. This dramatic increase can be explained by GG’s high water-holding capacity and its ability to form a three-dimensional gel network around soil grains. These water-trapping and gel-network formation mechanisms are also evident in the SEM images in Figure 6. In the SEM image of the ZS-GG4 sample shown in Figure 6B, the GG gel chains form a continuous, thick covering film (biopolymer film) throughout the mixture, restricting the hydraulic movement of water molecules and increasing the LL value by 190.7%. Similarly, the strong organic gel bridges formed in the calcite and quartz-rich RC-GG4 matrix (Figure 6B) and the biopolymer bindings that intercalate and clog the pores in BC-GG4 (Figure 6B) prevent water from escaping the matrix even when the water content drops to critical levels. This formation makes it difficult for soils to transition to the plastic and semi-solid phases, constituting the fundamental physical reason for the extraordinary increase in LL and PI values in all soil types. In the literature, many researchers state that the main reasons for the increase in LL values are increased liquid viscosity, aggregation, and soil–biopolymer interactions [7,10,32,33,40,52,53]. In GG-based mixtures, the biopolymer content and amount, rather than the soil type, are effective.

#### 3.4.3. Results of Compaction Characteristics (MDD and OMC)

**Figure 9:**
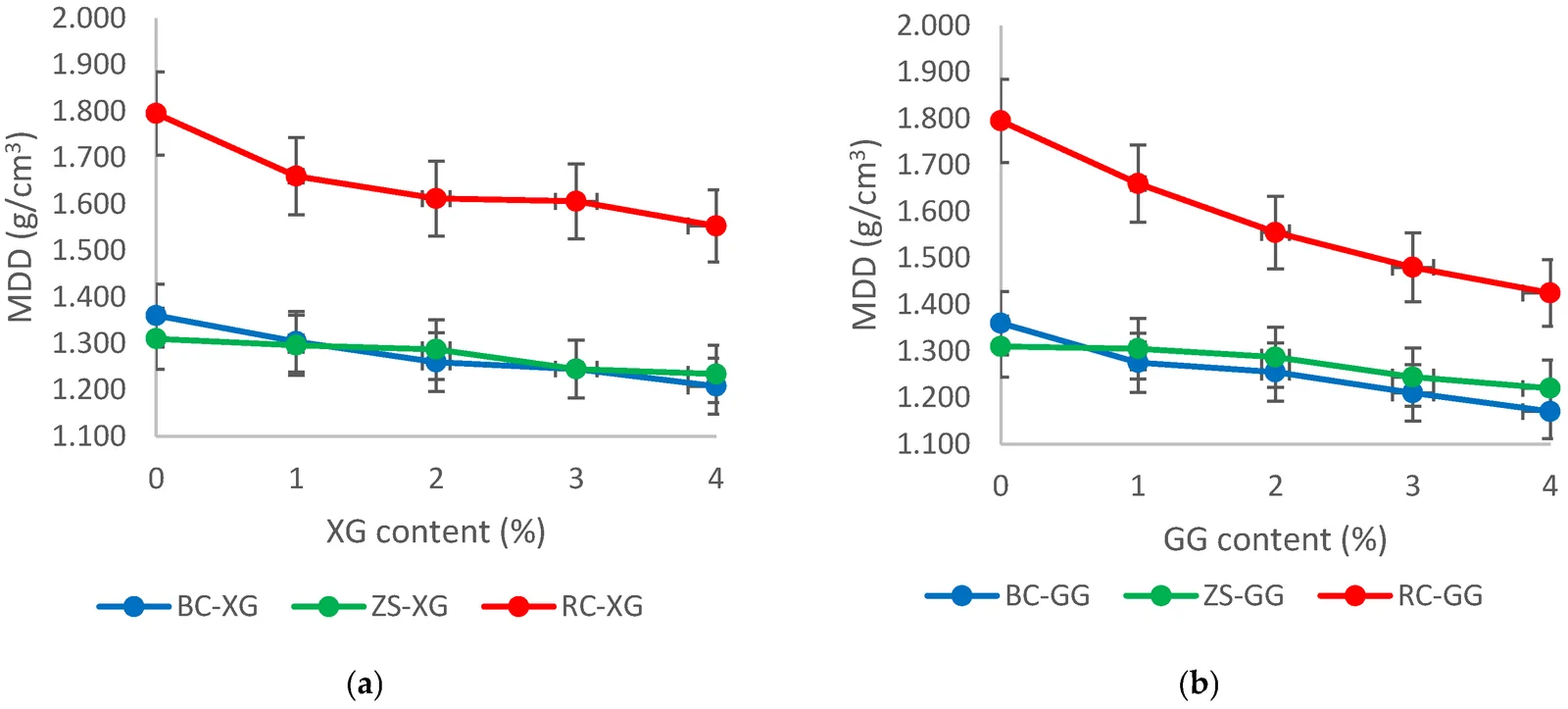

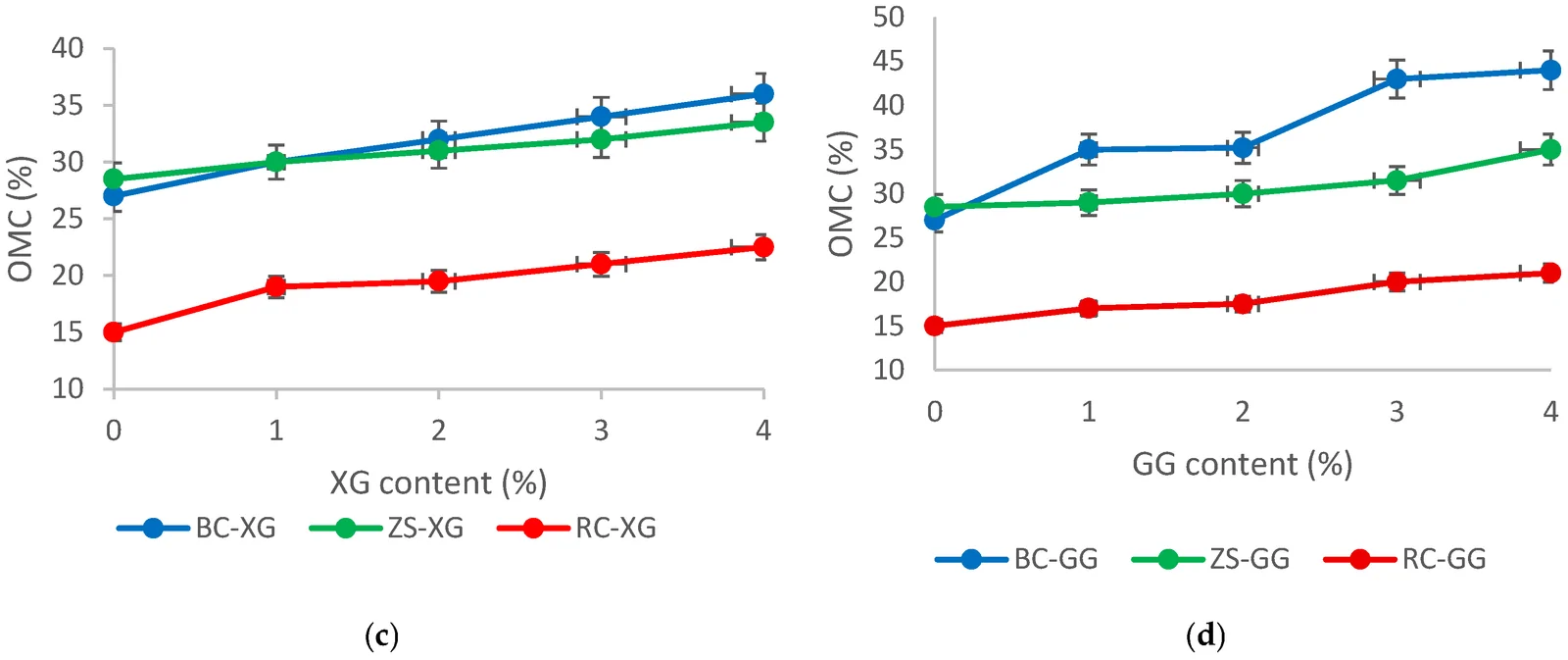
Variation in compaction properties with biopolymer content: (**a**) MDD for XG mixtures, (**b**) MDD for GG mixtures, (**c**) OMC for XG mixtures, (**d**) OMC for GG mixtures.

**Figure 10:**
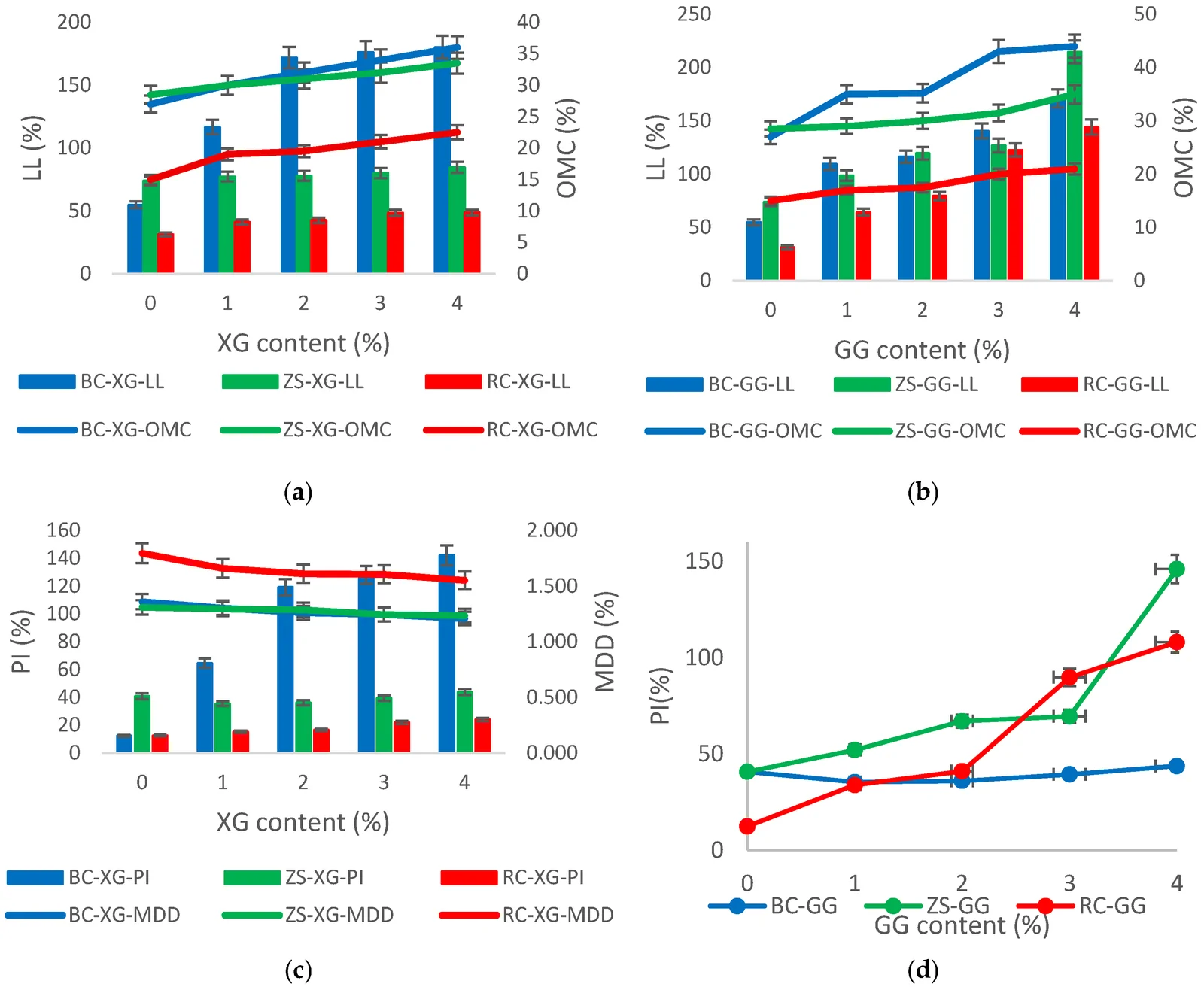
Relation of compaction and plasticity between mixtures ((**a**). OMC and PI in XG (**b**). OMC and LL in GG (**c**). MDD and PI in XG (**d**). MDD and PI in GG).

The compaction test results of the mixture samples are presented in Figure 9, and the relationship of these results with plasticity limits is presented in Figure 10. In mixtures made with XG, a clear decrease in MDD values was observed in all soil types (Figure 9a). In XG4 content, compared to the reference samples, a decrease in MDD values of 11.2% was obtained in BC mixtures, 5.8% in ZS mixtures, and 13.5% in RC mixtures. In OMC values, an increase of 33.3% was observed in BC mixtures, 17.5% in ZS mixtures, and 50.0% in RC mixtures (Figure 9c).

GG addition generally led to a greater decrease in MDD than XG (Figure 9b). Compared to the reference samples (GG0), MDD values in GG4 content decreased by 14.0% in BC mixtures, 6.9% in ZS mixtures, and 20.6% in RC mixtures. In terms of OMC values, an increase of 63.0% was detected in BC mixtures, 22.8% in ZS mixtures, and 40.0% in RC mixtures (Figure 9d). This classic “MDD decreases, OMC increases” trend, which is evident with biopolymer addition, is explained by microstructural and hydrophilic mechanisms occurring at the soil–biopolymer interface:

- Lubrication and Coating Effect: The high viscosity structure of GG, in particular, creates a slippery interface around the soil grains, making compaction difficult and leading to looser packing (low MDD) [21,39]. This situation is corroborated by the covering biopolymer films and gel bridges surrounding the crystal aggregates in the ZS-GG4 SEM images and the RC-GG4 images (Figure 6B). This covering prevents the direct transfer of mechanical compression energy to the mineral grains, which is the main reason for the reduction in dry density.
- Water Retention Capacity and Pore Filling: Biopolymer gels trap a high amount of water molecules within the soil matrix. The need for additional water to reach optimal consistency during compaction explains the sharp increases in OMC [54]. In the BC-GG4 example in Figure 6B, the high-water-retention-capacity gel networks that infiltrate between the clay plates (intercalation) and accumulate in the filled pores in Figure 6B trap water within the system, thus increasing the OMC values. In addition, the fact that biopolymers bind the soil grains into larger artificial aggregates leads to the soil being compacted at a lower MDD, as in a coarser-grained, rougher material [16,35,54].

The compaction test results are consistent with the changes in index and plasticity properties. The increasing trend in OMC values in the mixtures (Figure 9c,d) is supported by the extraordinary increase in liquid limit (LL) values discussed in Figure 8. Similarly, as clearly seen in Figure 10, a linear decrease in MDD values was obtained as the water absorption and swelling capacity of the mixture increased, as the plasticity index (PI) values of the mixtures increased (Figure 10).

In conclusion, the biopolymer additive radically altered the physical structure and compaction behavior of the soils. While the GG additive showed a stable and dominant effect in increasing water retention and plasticity in all three soil types thanks to the continuous gel films it formed in the microstructure, the effect of XG exhibited a more variable and specific behavior depending on the mineralogical type of the soil matrix.

### 3.5. Results of Shear Strength and Hydraulic Conductivity Tests

Shear strength and hydraulic conductivity test results for mixture samples are given in Table 5.

#### 3.5.1. Results of Shear Strength Tests

**Figure 11:**
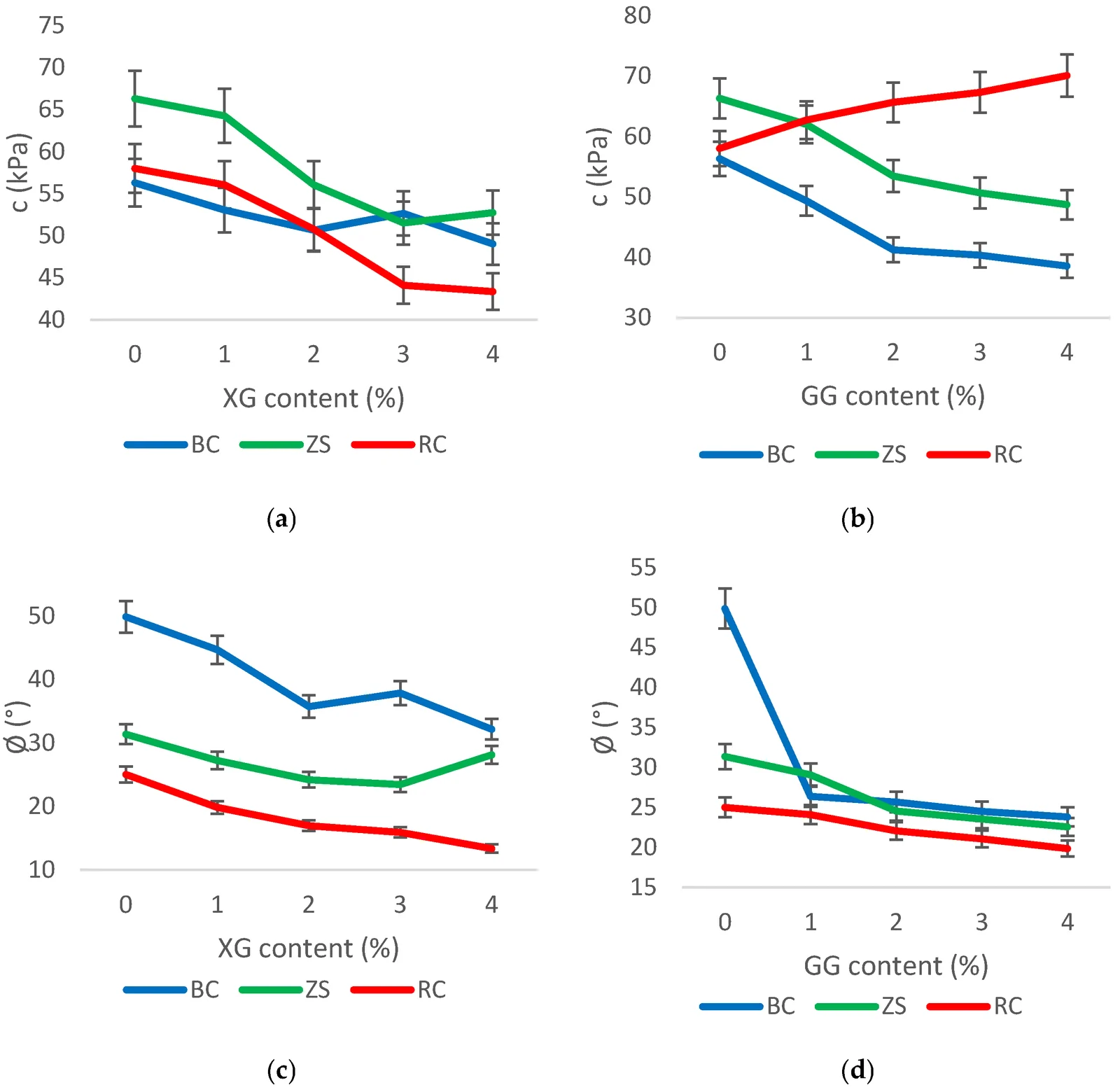
Shear strength parameters of mixtures ((**a**). c for XG; (**b**). c for GG; (**c**). Ø for XG; (**d**). Ø for GG mixtures).

Shear strength test results revealed that biopolymer mixtures altered the mechanical behavior of soils. The findings showed that the direction and magnitude of this change depended on the complex interaction between the biopolymer type, dosage, and soil type. Overall, the addition of XG caused a dose-dependent decrease in cohesion (c) and internal friction angle (Ø) in all tested soil types, while the addition of GG reduced cohesion in BC and ZS but increased it in RC. In contrast, the attenuating effect of GG on Ø was observed in all soil types (Figure 11). Below, the effects of XG and GG are evaluated separately for each soil type.

The decrease in c and Ø values in mixtures with XG can be attributed to the hydrogel network formed by XG, a water-absorbing polymer within the soil pores [55]. In soils with naturally high cohesion, such as BC and ZS, it is thought that the formed hydrogel penetrates between the soil particles, exhibiting a diluting and separating effect. Instead of forming strong, reinforcing bridges between soil particles, excessive hydrogel formation separates the particles, disrupting the soil’s natural structure and electrochemical bonds [15,34]. This results in a soil mixture with weakened natural cohesion. The viscous hydrogel coating formed on the soil particles acts as a lubricant, reducing particle-to-particle friction and interlocking, thereby decreasing Ø values. These findings are consistent with studies on improved sands and the literature showing that bond continuity is disrupted when the optimum biopolymer content is exceeded [7,56,57]. This weakening XG effect represents a mechanism diametrically opposed to the strengthening effect that GG creates in RC, detailed below.

##### Shear Strength Characteristics of Bentonite Clay (BC)

The addition of XG to BC mixtures resulted in a dose-dependent decrease in c and Ø values. In BC-XG4, the c value decreased by 13% compared to the reference sample (BC-XG0), falling from 56.3 kPa to 49.0 kPa, while the decrease in Ø (35%, from 49.9° to 32.2°) was much more pronounced. The addition of GG led to an even more dramatic decrease in BC. In BC-GG4, the c value decreased by 31.5% (from 56.30 kPa to 38.55 kPa), and the Ø value decreased by 52.3% (from 49.87° to 23.81°). These findings indicate that both biopolymers reduce the mechanical parameters of BC, with GG having a much stronger attenuating effect than XG.

##### Shear Strength Characteristics of Zeolite Sity Soil

In ZS-XG mixtures, the c value decreased by 26% in ZS-XG4 content compared to the reference sample (ZS-XG0), from 66.3 kPa to 52.7 kPa, and the Ø value decreased by 11.5%, from 31.37° to 28.13°. The slight increase in c value (from 51.50 kPa to 52.73 kPa) and the recovery in Ø value (from 23.45° to 28.13°) when switching from ZS-XG3 to ZS-XG4 suggest that the gel network may restructure at an excessive biopolymer dose (4%), resulting in partial mechanical improvement [57].

A similar weakening trend was observed in ZS-GG mixtures. The c value decreased by 26.5% in ZS-GG4 content, from 66.30 kPa to 48.70 kPa, while the Ø value decreased by 28.1%, from 31.37° to 22.57°. In ZS, the attenuating effect of GG was more balanced on c and Ø compared to XG, but more destructive on Ø.

##### Shear Strength Characteristics of Red Clay

In RC soil, the two biopolymers exhibited diametrically opposed behaviors. XG addition showed an attenuating effect, as in BC and ZS; in RC-XG4, the c value decreased by 25% (from 58.0 kPa to 43.4 kPa), and the Ø value decreased by 47% (from 25.02° to 13.35°). In contrast, GG addition produced a completely different and unexpected behavior in RC. The cohesion (c) value increased continuously with GG dosage; in RC-GG4, it increased by 20.8% relative to the reference sample, from 58.00 kPa to 70.05 kPa. This increase can be attributed to the strong hydrogen bonds formed between the hydroxyl groups of GG and the surface chemistry of RC (high Fe_2_O_3_ and MgO content according to XRF results). This interpretation is directly supported by the continuous, thick biopolymer networks surrounding the particles, which are clearly visible in the RC-GG4 SEM image in Figure 6B. In contrast, the effect of GG on the Ø value was attenuating, as with other soils, and decreased by 20.6% in RC-GG4 content, from 25.02° to 19.87°.

The GG effect is more complex. The gel structure formed by GG, with its high water-holding capacity, reduced intergranular friction across all soils, thereby decreasing Ø values. However, the effect of GG on cohesion (c) is critically dependent on the soil mineralogy. In soils such as BC and ZS, GG reduced cohesion similarly to XG; while in RC, which differs in surface chemistry (high Fe and Mg oxide content), it significantly increased cohesion by forming strong hydrogen bonds. This stark contrast between the weakening XG effect and the strengthening GG-RC effect clearly reveals the decisive role of soil mineralogy in biopolymer selection. The results of this study show that detailed mineralogical and chemical characterization of the soil to be used is essential for the effective and predictable application of biopolymer additives in soil improvement.

#### 3.5.2. Results of Hydraulic Conductivity (k) Tests

**Figure 12:**
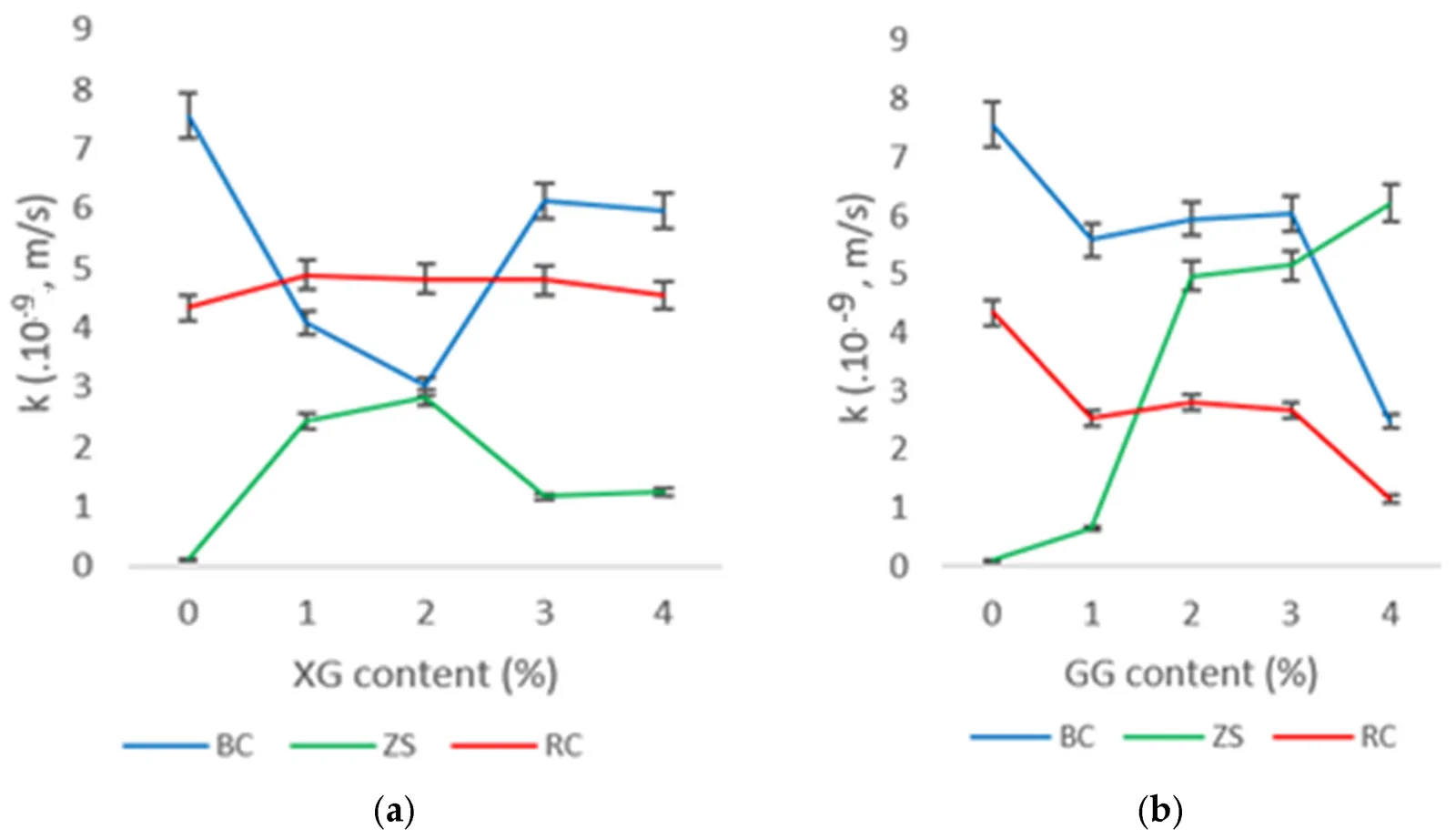
Hydraulic variation conductivity (k) with biopolymer content in (**a**) XG and (**b**) GG mixtures for different soils (BC, ZS, RC).

The k values of soil–biopolymer mixtures show that the addition of biopolymers can significantly alter the infiltration behavior of soils, and this effect is largely system-specific (Figure 12).

The effect of biopolymers on hydraulic conductivity is explained by two main mechanisms: the physical clogging of pores with biopolymer gels (pore-filling effect) and the lengthening of water passage paths by biopolymer films coating the grain surfaces (surface coating effect) [57,58,59]. The extent to which these mechanisms are effective varies depending on the type of biopolymer, its viscosity, and the soil mineralogy [60].

##### Hydraulic Conductivity Characteristics of Bentonite Clay (BC)

The effect of XG on BC exhibited a dose-dependent, non-systemic behavior. At low XG dosages (1–2%), a significant decrease in hydraulic conductivity was observed due to physical pore filling by the biopolymer gel. In BC-XG2, the k value decreased by approximately 60% compared to the reference sample (BC-XG0, 7.56 × 10^−9^ m/s), falling to 3.02 × 10^−9^ m/s. However, at high XG dosages (3–4%), excessive hydrogel formation separated the particles, resulting in a looser microstructure and fluctuations in hydraulic conductivity (BC-XG3: 6.13 × 10^−9^ m/s; BC-XG4: 5.95 × 10^−9^ m/s). This indicates that the effect of XG on BC is due to pore clogging at low doses and particle separation at high doses.

The addition of GG resulted in a more pronounced, dose-dependent decrease in hydraulic conductivity in BC. In BC-GG4, the k value decreased by approximately 67% compared to the reference sample, falling to 2.48 × 10^−9^ m/s. The high water-holding capacity of GG physically impedes water flow by forming a strong, cohesive hydrogel network within soil pores (pore blocking). The dramatic increases observed in LL and OMC in BC-GG mixtures confirm extensive water absorption and swelling of GG, indicating that this swelling reduces effective porosity and thus hydraulic conductivity [32,54].

##### Hydraulic Conductivity Characteristics of Zeolite Silty Soil (ZS)

The effect of XG was observed most clearly and strikingly in ZS. The zeolite initially has the lowest hydraulic conductivity value (0.107 × 10^−9^ m/s for ZS-XG0). With the addition of XG, the hydraulic conductivity increased by approximately 26-fold to 2.83 × 10^−9^ m/s (ZS-XG2). This finding is contrary to common reports in the literature that XG generally reduces permeability and highlights the unique interaction of XG with the porous zeolitic structure [25,27]. This behavior can be explained by the Donnan electrostatic exclusion effect. Anionic XG chains experience electrostatic repulsion from the dominant negative surface charge (permanent structural charge) of the zeolite. This repulsive force physically prevents high molecular weight XG polymers from entering the rigid micropores (crystal lattice) of the zeolite. Instead, the XG chains only coat the outer surfaces of the zeolite aggregates, forming a thin, slippery film layer (surface coating). During compaction, this coating reduces interaggregate friction, preventing tight particle packing; this is consistent with the observed decrease in MDD. As a result, a more open, interconnected, and continuous macropore network forms between the aggregates. This network provides a direct and low-friction path (macroflow path) for water flow, resulting in a dramatic increase in hydraulic conductivity of approximately 26-fold. This finding clearly demonstrates that XG’s effect on permeability is critically dependent on the soil’s mineralogical structure.

In contrast, the addition of GG increased the hydraulic conductivity in ZS in a dose-dependent manner. In ZS-GG4, the k value increased significantly compared to the reference sample (ZS-GG0, 0.107 × 10^−9^ m/s), reaching 6.22 × 10^−9^ m/s. This increase can be attributed to the high water-holding capacity of the GG gel structure, which causes the zeolite aggregates to swell, thereby opening interparticle voids and widening water-flow pathways. Furthermore, the increases in LL and OMC and the decrease in MDD in ZS-GG mixtures indicate that the mixture has transitioned to a looser structure with higher water content, which facilitates hydraulic conductivity.

##### Hydraulic Conductivity Characteristics of Red Clay (RC)

The addition of XG did not create a significant trend in RC. In RC-XG mixtures, the k value showed a slight increase in XG1 (4.88 × 10^−9^ m/s) compared to the reference sample (RC-XG0, 4.34 × 10^−9^ m/s), followed by a gradual decrease (RC-XG4: 4.53 × 10^−9^ m/s). This unsystematic behavior indicates that XG could not produce a net effect due to the presence of pore-filling and surface-coating mechanisms within the massive, dense aggregate structure of RC. The limited effect of XG on RC can be attributed to RC’s low plasticity (CL) and more densely packed structure.

In RC-GG mixtures, the permeability-reducing effect of GG reached its highest level. As the GG dosage increased, the hydraulic conductivity decreased continuously, and the k value in RC-GG4 decreased by approximately 74% relative to the reference sample, to 1.15 × 10^−9^ m/s. This most powerful effect stems from a combination of two mechanisms: (i) pore-filling by the GG gel and (ii) surface coating of GG, forming a continuous, dense, and low-permeability polymer film layer on the surface of RC particles, as clearly seen in the RC-GG4 SEM image in Figure 6B. This secondary mechanism is enabled by the strong hydrogen bonds formed between the hydroxyl groups of GG and the surface of the RC, which has high Fe_2_O_3_ and MgO contents. The remarkable increases in LL and PI and the significant decrease in MDD observed in RC-GG mixtures support this effect.

### 3.6. Comparative Evaluation of Cohesion Responses: The Role of Polymer Molecular Structure and Soil Surface Chemistry

The shear strength test results (Section 3.5.1) revealed a striking duality in the cohesion (c) response of soils to XG and GG additives. While XG consistently reduced cohesion in all soil types due to the formation of an attenuating hydrogel (as discussed), the effect of GG proved to be highly dependent on soil mineralogy. GG reduced cohesion in BC and ZS, but conversely increased it in RC. This contrasting behavior can be attributed to differences in the molecular structure of the biopolymers and the surface chemistry of the soils.

XG has a linear, rigid, rod-like structure in solution. This geometry allows it to form a continuous but slippery hydrogel film covering the particle surfaces, thus effectively limiting direct interparticle contact and disrupting the soil’s natural electrochemical bonds. This “lubricating” or “diluting” effect is independent of mineralogy and explains the universal reduction in cohesion in all BC-XG, ZS-XG, and RC-XG mixtures.

In contrast, GG has a highly branched, flexible, and helical-like structure. This architecture is less effective at forming a cohesive film, but is quite capable of forming multiple hydrogen bonds with active surface regions. In BC (montmorillonite-rich) and ZS (clinoptilolite-rich), the hydrophilic GG chains interact strongly with water, forming a thick hydration shell that increases the interparticle distance (steric hindrance). This separation force, combined with the gel’s lubricating effect, overcomes potential bridging and leads to a net reduction in cohesion.

However, the surface chemistry in RC is significantly different. XRF analysis (Figure 4c) showed that RC is rich in trivalent iron (Fe_2_O_3_, 7.49%) and magnesium (MgO, 12.99%) oxides. These metal oxides form high-energy, positively charged regions on the clay surface at typical soil pH. The abundant hydroxyl (-OH) groups in the branched chains of GG form strong, multiple hydrogen bonds with these Fe^3+^ and Mg^2+^ surface regions. This “polymer bridging” mechanism effectively cross-links the GG chains and RC particles, creating a robust, interconnected network that enhances the soil’s structural integrity. This micro-mechanism is visually confirmed in the SEM image of the RC-GG4 sample (Figure 6(B)c), where dense, continuous polymer bridges and gel networks are observed, tightly binding the hard crystalline aggregates together. This strong hydrogen bonding network is the primary reason for the 20.8% increase in cohesion observed in RC-GG4, highlighting the critical importance of clay surface chemistry in determining the success of biopolymer stabilization.

## 4. Conclusions

In this study, the effects of Xanthan Gum (XG) and Guar Gum (GG) biopolymers with different molecular geometries on three soil types (BC, ZS, RC) with different mineralogical structures were systematically investigated using macro-engineering experiments and micro-analytical methods (XRD, XRF, SEM). Based on the experimental and theoretical findings, the following main conclusions were reached:

This study systematically investigated the effects of Xanthan Gum (XG) and Guar Gum (GG) biopolymers on the geotechnical properties of three mineralogically distinct soils (Bentonite-BC, Zeolite silty soil-ZS, Red Clay-RC). Based on extensive experimental and analytical findings, the following specific conclusions and engineering recommendations were presented:

A. Mineralogy-Dependent Behavior: The effectiveness of biopolymers is critically dependent on the soil mineralogy and surface chemistry.

BC (Montmorillonite-rich): High plasticity and swelling potential led to excessive hydrogel formation, weakening mechanical properties.

ZS (Clinoptilolite-quartz-rich): The porous structure created a unique response, particularly in terms of hydraulic conductivity.

RC (Calcite-quartz-rich, high Fe_2_O_3_/MgO): High metal oxide content facilitated the formation of strong chemical bonds with GG.

B. Compression and Index Properties: Both biopolymers generally reduced MDD and increased plasticity limits with OMC.

Key Findings: The specific gravity (Gs) of RC exhibited a nonlinear response: it decreased at low doses (dilution) and increased at high doses (chain entanglement and flocculation).

C. Shear Strength (Cohesion, c): XG; showed a lubricating and insulating effect by reducing cohesion (c) and friction angle (φ) in all soil types. Not recommended for applications requiring high shear strength.

GG; exhibited bidirectional behavior. Reduced cohesion in BC and ZS due to steric hindrance, but significantly increased cohesion in RC by 20.8% (from 58.00 to 70.05 kPa) due to strong hydrogen bonding with surface iron and magnesium oxides.

D. Hydraulic Conductivity (k): XG; unexpectedly increased the hydraulic conductivity of ZS by approximately 26-fold. This anomaly is attributed to the Donnan electrostatic exclusion effect, in which anionic XG chains are repelled from the zeolite surface, thereby creating open macro-flow channels. This finding challenges the general assumption that XG reduces permeability.

GG; effectively reduced hydraulic conductivity in all soil types through pore filling and surface coating. The most significant reduction (74%) was achieved in the RC-GG4 mixture.

E. Engineering Application Recommendations:

For Seepage Barriers and Landfill Liners: The RC-GG4 mixture is the most suitable candidate. Offering a unique combination of increased shear strength (high stability) and significantly reduced hydraulic conductivity (high impermeability), it provides an environmentally friendly alternative for lining applications. (This mixture is recommended for high-strength and low-cost projects).

For Drainage Applications: ZS-XG mixtures are not suitable for barriers due to their significantly increased hydraulic conductivity, but offer a potential geotechnical solution for drainage layers or high-permeability filters where the risk of clogging by conventional materials is a concern. (This mixture is recommended for high-amplitude drainage applications).

For General Stabilization: In BC and ZS soils, neither XG nor GG addition increased shear strength. Therefore, the use of these biopolymers for strength-based stabilization of these specific soil types without further optimization or combination with other additives is not recommended. (In BC and ZS soils, since these biopolymers do not increase strength, their use alone in the improvement of these soils is not recommended).

While this study provides important insights into the fundamental interaction mechanisms between biopolymers and different soil minerals, it also has some limitations. Firstly, the study was conducted under controlled laboratory conditions on remolded and homogenized samples. The performance of the most promising blends (e.g., RC-GG4) should be validated in the field under varying environmental and loading conditions. Secondly, this study primarily focused on short-term static engineering properties. The long-term durability of biopolymer-soil matrices under extreme conditions such as soak-dry cycles, freeze–thaw cycles, and chemical attack has not been investigated.

Therefore, future research should focus on: (1) evaluating the long-term durability and aging properties of selected high-performance composites under cyclic environmental loading; (2) conducting large-scale field trials to validate laboratory findings; and (3) investigating the potential interaction of biopolymers with other environmentally friendly additives (e.g., fibers, industrial by-products) to further optimize mechanical and hydraulic performance for a wider range of geotechnical applications.

## Figures and Tables

**Table 1:** Basic properties of XG and GG biopolymers.

Properties	XG	GG	References
Common source	Xanthomonas compestris (bacterium)	Cyamopsis tetragonoloba (plant)	[9,28,29,30,31]
Chemical Nature	Anionic heteropolysaccharide	Non-ionic galactomannan	[9,29,30,31]
Molecular weight	0.9–1.6 × 10^6^	<2 × 10^6^	[32]
pH	7.03	6.5	[10,31]
Plasticity	Pseudoplastic	Pseudoplastic	[29,32]

**Table 2:** Sample mixture ratios and symbols used.

Sample Name	Soils Content (%)			XG (%)	Sample Name	Soils Content (%)			GG (%)
	BC	ZS	RC			BC	ZS	RC	
BC-XG0	100			0	BC-GG0	100			0
BC-XG1	99			1	BC-GG1	99			1
BC-XG2	98			2	BC-GG2	98			2
BC-XG3	97			3	BC-GG3	97			3
BC-XG4	96			4	BC-GG4	96			4
ZS-XG0		100		0	ZS-GG0		100		0
ZS-XG1		99		1	ZS-GG1		99		1
ZS-XG2		98		2	ZSGG2		98		2
ZS-XG3		97		3	ZS-GG3		97		3
ZS-XG4		96		4	ZS-GG4		96		4
RC-XG0			100	0	RC-GG0			100	0
RC-XG1			99	1	RC-GG1			99	1
RC-XG2			98	2	RC-GG2			98	2
RC-XG3			97	3	RC-GG3			97	3
RC-XG4			96	4	RC-GG4			96	4

**Table 3:** Geotechnical properties of soils.

Properties	BC	ZS	RC
Fine (%)	94.55	61.25	96.87
Sand (%)	5.01	38.38	2.99
Gravel (%)	0.44	0.37	0.14
Specific gravity (Gs)	2.447	1.740	2.550
Liquid Limit (LL, %)	54.86	73.87	31.27
Plasticity Index (PI, %)	12.25	40.76	12.43
Soil Classification (USCS)	MH	CH	CL
Optimum moisture content (OMC, %)	27.00	28.50	15.00
Maximum dry density (MDD, g/cm^3^)	1.360	1.310	1.795
Cohesion (c, kPa)	56.30	66.30	58.00
Internal friction angle (Ø, °)	49.87	31.37	25.02
Hydraulic conductivity (k, m/s)	7.56 × 10^−9^	0.107 × 10^−9^	4.34 × 10^−9^

**Table 4:** Index and compaction properties of mixtures.

Mixture	Gs	LL (%)	PI (%)	MDD (g/cm^3^)	OMC (%)
BC-XG0	2.447	54.86	12.25	1.360	27.00
BC-XG1	2.324	116.68	64.67	1.304	30.00
BC-XG2	2.226	171.88	119.20	1.260	32.00
BC-XG3	1.923	176.27	128.15	1.245	34.00
BC-XG4	1.883	180.21	142.18	1.208	36.00
BC-GG0	2.447	54.86	12.25	1.360	27.00
BC-GG1	2.315	109.51	48.75	1.275	35.00
BC-GG2	2.289	116.41	48.00	1.255	35.20
BC-GG3	2.241	140.54	74.62	1.210	43.00
BC-GG4	2.186	171.01	111.86	1.170	44.00
ZS-XG0	1.740	73.87	40.76	1.310	28.50
ZSXG1	1.962	77.35	35.43	1.296	30.00
ZS-XG2	1.775	77.83	36.05	1.287	31.00
ZSXG3	1.710	80.00	39.39	1.245	32.00
ZS-XG4	1.440	84.67	43.81	1.234	33.50
ZS-GG0	1.740	73.87	40.76	1.310	28.50
ZS-GG1	2.023	98.72	52.13	1.305	29.00
ZS-GG2	1.924	119.64	67.05	1.287	30.00
ZS-GG3	1.852	126.84	69.50	1.244	31.50
ZS-GG4	1.764	214.70	146.03	1.220	35.00
RC-XG0	2.550	31.28	12.43	1.795	15.00
RC-XG1	2.413	41.06	15.19	1.660	19.00
RC-XG2	1.979	42.45	16.63	1.612	19.50
RC-XG3	2.342	48.46	21.86	1.606	21.00
RC-XG4	2.419	48.65	23.99	1.553	22.50
RC-GG0	2.550	31.28	12.43	1.795	15.00
RC-GG1	2.324	64.33	33.89	1.660	17.00
RC-GG2	2.226	79.42	41.08	1.555	17.50
RC-GG3	2.342	122.62	89.83	1.480	20.00
RC-GG4	2.419	144.19	108.07	1.425	21.00

**Table 5:** Shear strength and hydraulic conductivities of mixtures.

Mixture	c (kPa)	Ø (°)	k (×10^−9^, m/s)
BC-XG0	56.30	49.87	7.56
BC-XG1	53.05	44.66	4.08
BC-XG2	50.65	35.74	3.02
BC-XG3	52.65	37.86	6.13
BC-XG4	49.00	32.16	5.95
BC-GG0	56.30	49.87	7.56
BC-GG1	49.35	26.40	5.59
BC-GG2	41.25	25.68	5.95
BC-GG3	40.35	24.51	6.04
BC-GG4	38.55	23.81	2.48
ZS-XG0	66.30	31.37	0.11
ZS-XG1	64.25	27.24	2.43
ZS-XG2	56.05	24.20	2.83
ZS-XG3	51.50	23.45	1.16
ZS-XG4	52.73	28.13	1.24
ZS-GG0	66.30	31.37	0.11
ZS-GG1	62.00	29.07	0.65
ZS-GG2	53.45	24.56	4.97
ZS-GG3	50.65	23.57	5.15
ZS-GG4	48.70	22.57	6.22
RC-XG0	58.00	25.02	4.34
RC-XG1	56.05	19.84	4.88
RC-XG2	50.75	16.95	4.82
RC-XG3	44.10	15.91	4.79
RC-XG4	43.35	13.35	4.53
RC-GG0	58.00	25.02	4.34
RC-GG1	62.70	24.11	2.53
RC-GG2	65.65	22.08	2.81
RC-GG3	67.30	21.07	2.66
RC-GG4	70.05	19.87	1.15

## Data Availability

The data presented in this study are available on request from the corresponding author due to privacy.
